# Covalent Functionalization of MXenes with Porphyrin for Visible‐Light Activation in Energy Conversion Nanodevices

**DOI:** 10.1002/smll.202503895

**Published:** 2025-08-03

**Authors:** Hong Li, Ruben Canton‐Vitoria, Yuto Urano, Sudhanshu Kumar Nayak, Eisuke Yamamoto, Makoto Kobayashi, Ryo Kitaura, Minoru Osada

**Affiliations:** ^1^ Department of Materials Chemistry & Institute of Materials and Systems for Sustainability (IMaSS) Nagoya University Nagoya 464–8601 Japan; ^2^ Joining and Welding Research Institute Osaka University Osaka 567‐0047 Japan; ^3^ International Center for Materials Nanoarchitectonics National Institute for Materials Science 1‐1 Namiki Tsukuba 305‐0044 Japan; ^4^ Ultrafast Photophysics and Photonics Laboratory Department of Physics Indian Institute of Technology Hyderabad Telangana Kandi 502285 India; ^5^ Research Institute for Quantum and Chemical Innovation Institutes of Innovation for Future Society Nagoya University Nagoya 464–8601 Japan

**Keywords:** 2D Materials, covalent functionalization, light conversion, MXenes, photosensors

## Abstract

MXenes such as Ti_3_C_2_ and Ti_3_CN are 2D materials characterized by the presence of a T*_x_* phase that passivates the reactive titanium surface. Modifying their chemical composition to anchor target molecules is of great interest for addressing key challenges, such as enhancing the conversion of visible light into electricity. In this study, a covalent functionalization strategy is developed to modify the T*_x_* phase of Ti_3_C_2_ or Ti_3_CN with alkyl amines, followed by coupling with Zn‐porphyrin. This process activates the optical properties of MXenes without causing any damage. X‐ray photoelectron spectroscopy and infrared spectroscopy are pivotal in confirming the covalent functionalization, while thermogravimetric analysis, transmission electron microscopy, and additional techniques provided further insights into structural and chemical features. Spectroelectrochemical investigations reveal carrier injection into MXenes under light illumination, potentially enhancing conductivity. Photodetectors fabricated from these films demonstrate responsivities of 1.4–15.0 A W^−1^ and external quantum efficiencies ranging from 1300 to 2830% in the visible range, making them comparable to well‐established hybrid 2D nanomaterials like MoS_2_ and WS_2_.

## Graphical abstract

MXene surfaces are covalently functionalized with porphyrin, yielding hybrids that exhibit electron–hole transfer under light illumination and enable high‐quality nanofilm formation for advanced optical sensing.

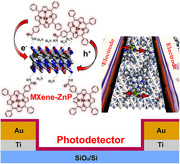

## 1 Introduction

MXenes represent a rapidly emerging class of 2D materials. Ti_3_C_2_, the most widely studied MXene, consists of three atomically thin titanium layers interleaved with two atomically thin carbon layers, each plane exhibiting a hexagonal honeycomb structure. Ti_3_C_2_ exhibits metallic behavior, characterized by a low recombination rate of photoactivated electron–hole (e^−^–h^+^) pairs^[^ ^1^ ^]^ and exceptional electrical conductivity of 2.4 × 10^6^ S m^−1^,^[^ ^2^, ^3^ ^]^ nearly twice that of graphene.^[^ ^4^, ^5^ ^]^ On the other hand, Ti_3_CN is semiconducting, with a bandgap of 1.16 eV^[^ ^6^ ^]^ and a conductivity of 2.5 × 10^5^ S m^−1^, millions of times greater^[^ ^7^, ^8^ ^]^ than that of MoSe_2_^[^ ^9^, ^10^ ^]^ or MoS_2_.^[^ ^11^ ^]^ MXenes are also known for their excellent solubility and rich surface chemistry, making them applicable across a wide range of fields, including energy storage^[^ ^12^, ^13^ ^]^ catalysis,^[^ ^14^, ^15^ ^]^ electromagnetic interference shielding,^[^ ^16^, ^17^ ^]^ medicine,^[^ ^18^, ^19^ ^]^ and electronics.^[^ ^20^, ^21^ ^]^

Modification of the MXene surface is of great interest, as it allows control over key properties. The T_x_ phase passivates the titanium planes and is easily tunable, enabling the presence of different functional groups such as ─OH, ─F, ─S, or ─N, which enrich the chemistry and capabilities of MXenes. Covalent functionalization offers the advantage of creating a robust connection between 2D nanomaterials and target components, enabling atomic‐level contact between the two species. Unlike other 2D materials such as graphene, where sp^2^ carbon must be converted to sp^3^ during chemical modification,^[^ ^22^ ^]^ the T_x_ phase of MXenes allows for covalent functionalization without damaging the basal plane. However, the covalent modification of MXenes is limited to five main reactions: phosphate,^[^ ^23^, ^24^ ^]^ carboxylate,^[^ ^25^, ^26^ ^]^ silane,^[^ ^27^, ^28^ ^]^ diazonium salt,^[^ ^29^, ^30^ ^]^ and amination,^[^ ^31^ ^]^ in addition to polymerization reactions.^[^ ^32^, ^33^ ^]^ While these approaches are well‐established, a lack of simple characterization techniques that can definitively confirm the formation of covalent bonds remains a challenge. Indeed, the new Ti─O bonds are difficult to distinguish from those already present in the T_x_ phase in techniques such as X‐ray photoelectron spectroscopy (XPS), necessitating the use of more advanced techniques as high‐resolution transmission electron microscopy (TEM). Only amination, a recently developed technique, has been shown to form a distinguishable Ti─N bond while preserving the surface of MXenes, therefore facilitating the use of straightforward characterization techniques.^[^ ^31^ ^]^ However, the potential of diamines has not yet been fully exploited. The bifunctionality of diamines provides anchoring points for more complex functional groups, such as chromophores, enabling precise control over MXenes' optoelectronic properties.

In its pure form, Ti_3_C_2_ and Ti_3_CN are not optically active. However, slight modifications, such as controlling the functional groups in the T_x_ layer with groups like ─OH or ─F, can open an indirect bandgap in Ti_3_C_2_ and tune that of Ti_3_CN.^[^ ^1^, ^2^, ^3^ ^]^ Furthermore, hybridization with target inorganic materials can activate their photoactive properties.^[^ ^34^ ^]^ For example, Ti_3_C_2_ functionalized with TiO_2_ has achieved responsivities of 202 A W^−1^ under UV irradiation, but its response is limited under visible light.^[^ ^35^ ^]^ Other 2D materials, such as graphene, MoS_2_, or WS_2_, have also improved their optical performance after functionalization with chromophores like porphyrin.^[^ ^36^ ^]^ While light‐conversion applications,^[^ ^37^, ^38^, ^39^, ^40^ ^]^ like photocatalysis,^[^ ^41^, ^42^, ^43^ ^]^ have been explored, the development of MXenes‐based photosensors functionalized with photoactive molecules remains scarce. It is expected that chromophores will activate the optical properties of MXenes by transferring excitonic species and enhancing the number of carriers. Then, the electron–hole pairs will have a lower recombination ratio compared to materials with a direct bandgap. Hence, MXenes functionalized with chromophores might compete with other exceptional optically active 2D hybrids, such as transition metal dichalcogenides,^[^ ^44^, ^45^ ^]^ in the field of nanotechnology.

In this study, we investigate the covalent functionalization of Ti_3_C_2_ and Ti_3_CN by introducing alkyl diamines to the T*_x_* phase, which subsequently react with porphyrins via amidation to form photoactive hybrid materials. The loading of organic species is controlled using techniques like the Kaiser test and thermogravimetric analysis (TGA), while the covalent functionalization is confirmed by X‐ray photoelectron spectroscopy. Additionally, infrared (IR) spectroscopy confirms the formation of amide bonds and ensures the complete removal of non‐covalent species. X‐ray diffraction (XRD) demonstrates layer‐by‐layer functionalization, and TEM reveals the atomic structure. Optical and electrochemical studies show that charge carriers are introduced to Ti_3_C_2_ and Ti_3_CN through electron and hole transfer events from the porphyrins upon light irradiation, a key process explaining the exceptional performance of the resulting photodevices across the full visible spectrum.

## 2 Results and Discussion

### 2.1 Synthesis and Characterization

Ti_3_AlC_2_ and Ti_3_AlCN MAX phases were etched with ethane‐1,2‐diaminium difluoride salt mixed in HCl (12 m) for 12 h at 60 °C. During this process, F^−^ ions released from the nitrogen salts attack the aluminum of Ti_3_AlC_2_ and Ti_3_AlCN. As a result, the atomic Al layer undergoes oxidation, and hydrogen bubbles are observed. This etching process exposes an uncoordinated Ti layer, which can react with H_2_O molecules as well as with Cl^−^ or F^−^. Similar reactions have been observed in MAX phases and other molten salts like NH_4_F, LiF, or NaF.^[^ ^46^, ^47^, ^48^ ^]^ However, in this reaction, ethane‐1,2‐diaminium difluoride salt also releases amines that can further react with Ti. After filtration and pH‐neutralization with trimethylamine, the surfaces of the layered nanosheets Ti_3_C_2_T*_x_* (**a1**) and Ti_3_CNT*_x_* (**b1**) contain primary amines linked to the nanosheets, which are capable of undergoing typical organic chemistry reactions. The presence of the primary amines in Ti_3_C_2_T*_x_* and Ti_3_CNT*_x_* was evidenced by the Kaiser test, showing loadings of 141 and 120 µmol g^−1^, respectively. These Kaiser test values were notably high, consistent with other covalent functionalization techniques employed with graphene.^[^ ^49^ ^]^

**Figure 1:**
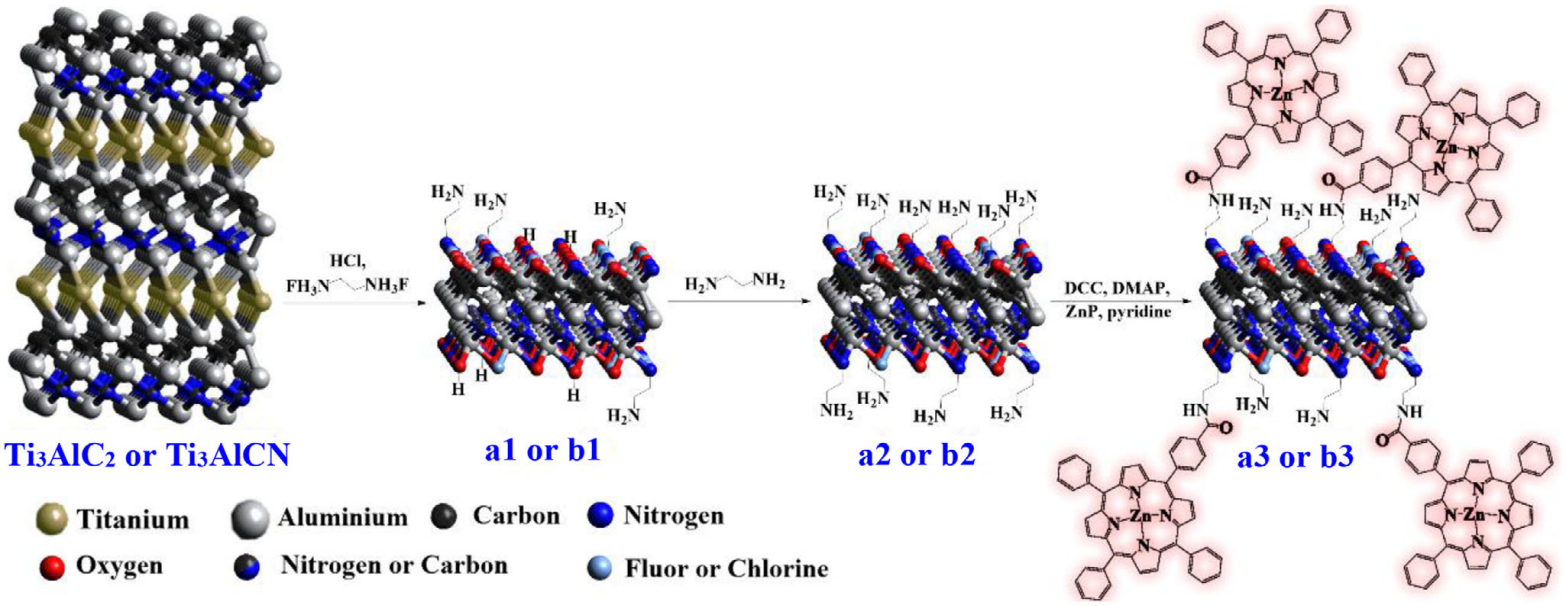
Reaction route for MXene **a3** and **b3** hybrid materials.

One of the main goals in this study was to achieve the highest possible amine loading to homogenize the T*_x_* layer and minimize secondary reactions in subsequent steps. Therefore, to increase the level of free amines, a second reaction was performed with ethylenediamine (EDA) at 75 °C, which served as both solvent and reactant (**Figure** **1**). Due to the greater stability of Ti‐amine bonds compared to Ti─OH, Ti─F, or Ti─Cl bonds,^[^ ^31^, ^50^ ^]^ the surface of the T*_x_* phase was exchanged to contain a larger number of free amines, resulting in Ti_3_C_2_T_NH2_ (**a2**) and Ti_3_CNT_NH2_ (**b2**), with Kaiser test values of 370 and 307 µmol g^‒1^, respectively. The amines on nanosheets **a2** and **b2** can react with the carboxylic acid group in target molecules, such as 5‐(4‐carboxyphenyl)‐10,15,20‐(triphenyl) porphyrin Zn^2+^ (ZnP), through amination. This reaction used *N*,*N*′‐Dicyclohexylcarbodiimide (DCC) and 4‐Dimethylaminopyridine (DMAP) as coupling agents, yielding the hybrid materials Ti_3_C_2_T_ZnP_ (**a3**) and Ti_3_CNT_ZnP_ (**b3**). The first evidence of functionalization came from the Kaiser test, which showed a decrease in amine loading from 370 and 307 µmol g^‒1^ in **a2** and **b2** to 40 and 72 µmol g^‒1^ in **a3** and **b3**, respectively (see Section 4 Experimental Section).

**Figure 2:**
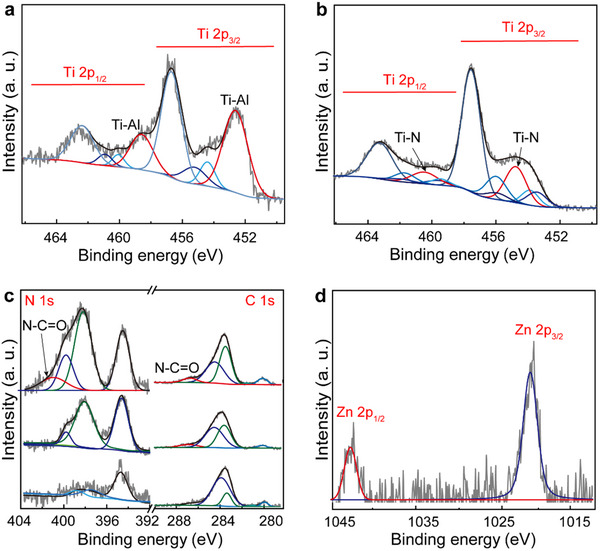
XPS spectra of Ti 2p for a) pristine Ti_3_AlCN and b) **b2**. c) N 1s (left) and C 1s (right) for pristine Ti_3_AlCN (bottom), **b2** (middle) and **b3** (top). d) Zn 2p for pristine Ti_3_AlCN **b3**.

XPS proved indispensable for characterizing the bonding nature of the materials, revealing the formation of Ti‒N bonds for **a2** and **b2**, and amide carbonyl groups for hybrid materials **a3** and **b3** (**Figure** **2**; Figure S1, Supporting Information). Initially, the Ti 2p spectra of Ti_3_AlCN can be deconvoluted into signatures at 462.55, 461.05, and 460.20 eV, corresponding to 2p_1/2_ levels.^[^ ^51^, ^52^ ^]^ These signatures can be assigned to various bonds, including Ti‒N and Ti‒C, all appearing as doublets in the 2p_3/2_ level, with binding energy separations of ≈5.65 eV, observed at 456.80, 455.25, and 454.50 eV, respectively. However, we focus on the signatures at 458.75 and 452.7 eV, which correspond to Ti‒Al bonds.^[^ ^53^ ^]^ In **a2** and **b2**, the Ti‒Al bands significantly decrease, ensuring the chemical etching while new Ti‒N signatures appear at 460.45 and 454.75 eV, suggesting the successful formation of a covalent bond between Ti and the amine (Figure 2a,b; Figure S1a,b, Supporting Information).^[^ ^31^ ^]^ No significant changes in the Ti 2p_1/2_ and 2p_3/2_ levels are observed when comparing **b2** and **b3** with **a3** or **b3** (Figure S2, Supporting Information). The identical spectra confirm that the Ti─N bond persists after the reaction, while the Ti─O band remains weak, indicating that no degradation occurred during the reaction process.

Next, the N 1s spectrum of Ti_3_AlCN shows negligible organic derivative signatures, with a peak at 393.45 eV corresponding to the graphenic nitrogen plane. The addition of amines in **b2** significantly increases the presence of NH and NH_2_ bonds at 398.35 and 400.01 eV, respectively, confirming the incorporation of EDA molecules (Figure 2c). Although direct evidence for covalent functionalization between the amines and Ti_3_CN via nitrogen is lacking, the low boiling point of EDA and the vacuum conditions of the XPS analysis (8 × 10^−8^ Torr) suggest sufficient interaction between the amines and Ti_3_CN to prevent evaporation during the XPS analysis. For Ti_3_C_2_ in **a2**, a weak but discernible band at 394.65 eV, similar to that observed in Ti_3_CN, appears, supporting the formation of a new Ti−N covalent bond (Figure S1c, Supporting Information).^[^ ^54^ ^]^ After the addition of porphyrin in **b3**, new bands corresponding to amide (N─C═O) bonds were recorded at 401.23 eV, confirming the success of the coupling reaction.^[^ ^55^ ^]^ The C 1s spectra of hybrid materials **a3** and **b3** were also relevant (Figure 2c; Figure S1c, Supporting Information). First, pristine Ti_3_AlC_2_ exhibits signatures at 283.5 and 279.9 eV, attributed to the presence of Ti─C─Ti and C─Ti─N bonding, which persist in the spectra of **a2** and **b2**.^[^ ^54^, ^56^ ^]^ These interactions are highly predominant, masking signatures associated with organic species. However, the emergence of a new band at 286.95 eV suggests the presence of an N─C═O bond, further supporting the covalent linkage between the amines and the porphyrin. Finally, the Zn 2p_1/2_ and Zn 2p_3/2_ binding energies at 1043.5 and 1020.3 eV, respectively, confirm the presence of ZnP in hybrid materials **b3** and **a3** (Figure 2d; Figure S1d, Supporting Information).

**Figure 3:**
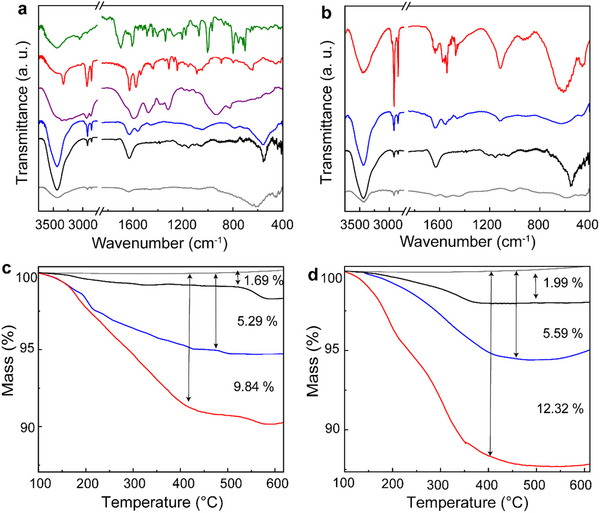
IR spectra for a) Ti_3_AlC_2_ (gray), **a1** (black), **a2** (blue), **a3** (red), EDA (purple), and ZnP (green), and b) Ti_3_AlCN (gray), **b1** (black), **b2** (blue), and **b3** (red). Thermographs for c) Ti_3_AlC_2_ (gray), **a1** (black), **a2** (blue), **a3** (red), and d) Ti_3_AlCN (gray), **b1** (black), **b2** (blue), and **b3** (red).

IR spectroscopy is highly useful for evaluating the organic moieties within hybrid materials (**Figure** **3**a,b). First, Ti_3_AlC_2_ and Ti_3_AlCN MAX phases showed negligible characteristic IR signatures, whereas the nanosheets **a1** and **b1** exhibited the moieties at 3455 and 3439 cm^−1^, associated with O─H and N─H vibrations, alongside the presence of C─H moieties at the 2700–3000 cm^−1^ range. The C─H signatures were more pronounced in **a2** and **b2**, as the O─H loading decreased in comparison with new alkyl amines. Additionally, new bands at 1557 and 1554 cm^−1^, characteristic of EDA‐like moieties, were observed. Hybrid materials **a3** and **b3** are further characterized by the presence of amide signatures, at 1630 and 1572 cm^−1^ for **a3** and 1632 and 1577 cm^−1^ for **b3**, providing conclusive evidence for covalent linkage with ZnP. The absence of the band at 1701 cm^‒1^, which corresponds to the carboxylic acid of pristine ZnP, confirms that the hybrid materials **a3** and **b3** are free of non‐covalent species.

Raman spectroscopy was used to analyze the vibronic features of the MXene nanosheets of **a1**–**3** and **b1**–**3** (Figure S3a,b, Supporting Information). Initially, the MAX phases showed no predominant peaks, whereas the nanosheets **a1** and **b1** exhibited similar signatures at 155, 259, 410, 608, 1338, 1592, 2976, 3229, and 3320 cm^−1^. The peak at 155 cm^−1^ corresponds to the doubly degenerated (E_g_) mode for the in‐plane surface Ti vibrations,^[^ ^57^, ^58^ ^]^ while the peak at 608 cm^−1^ is associated with Ti─C^[^ ^50^ ^]^ and Ti─N^[^ ^59^ ^]^ vibrations. In contrast, the peaks at 258 and 410 cm^−1^ are related to the T*_x_* layer,^[^ ^60^ ^]^ suggesting the presence of Ti─F, Ti─O^[^ ^60^ ^]^ and Ti─N^[^ ^7^ ^]^ bonds, and the peaks at 1338 and 1592 cm^−1^ are indicative of a carbon *pseudo*‐graphenic layer.^[^ ^61^, ^62^ ^]^ Finally, the bands between 2900 and 3400 cm^−1^ are attributed to the alkyl chain of amines.^[^ ^63^, ^64^ ^]^ The intensity of the alkyl chain peaks in **a2** and **b2**, compared to the E_g_ mode, is higher than in **a1** and **b1**, indicating a greater content of amines. The variations in intensity between the bands at 259–608 cm^−1^ in hybrid materials **a3** and **b3** may be due to modifications of the T*_x_* layer, such as the transformation from amine to amide and basal functionalization with ZnP. The absence of shifts in the E_g_ mode (155 cm^−1^) or any other signatures in hybrid materials **a3** or **b3** suggests minimal impact on the vibrational structure of Ti_3_C_2_T*_x_* and Ti_3_CNT*_x_* nanosheets.

The XRD patterns of Ti_3_AlC_2_ and Ti_3_AlCN (Figure S3c,d, Supporting Information) show peaks at 9.54° and 9.59°, which shift to lower angles in **a2** and **b2**, reaching angles of 6.44° and 6.28°. This suggests an expansion of the interlayer spacing. According to Bragg's formula, the interlayer spacing increases from 9.26 and 9.21 Å in Ti_3_AlC_2_ and Ti_3_AlCN to 13.71 and 13.62 Å in **a2** and **b2**, further expanding to 14.15 Å in both hybrid materials **a3** and **b3**. The slight increase in spacing of **a3** and **b3** indicates that the porphyrin skeleton is strongly attracted to the basal plane of the MXenes, maximizing the interaction between the π orbitals of the porphyrin, the d‐orbitals of Zn, and the d‐orbitals of Ti, both being perpendicular to the basal plane. This enhanced interaction is facilitated by the flexibility of the amines, allowing more intimate contact compared to other covalent functionalization methods, such as those performed via diazonium salts, in which porphyrins interact perpendicularly with the basal plane of the MXene, thereby limiting π–d orbital hybridization.^[^ ^29^, ^38^ ^]^

TGA was used to determine the loading of organic addends within all the functionalized materials (Figure 3c,d). The Ti_3_AlC_2_ and Ti_3_AlCN MAX phases remained stable in the temperature range of 100–600 °C under a nitrogen atmosphere. Nanosheets **a1** and **b1** showed a mass loss between 150 and 500 °C of 1.69% and 1.99%, respectively, which can be attributed to the degradation of the organic and oxygen species in the T*_x_* layer. The level of functionalization increases in **a2** and **b2**, with a mass loss of 5.29% and 5.59%, respectively, which further rises to 9.84% and 12.32% in hybrid materials **a3** and **b3**. These results indicate a relatively high degree of functionalization, with a value of one EDA‐like functional group per 6.4 and 5.9 units of Ti_3_C_2_ or Ti_3_CN for **a2** and **b2**, and one ZnP per 41.8 and 32.1 units of Ti_3_C_2_ or Ti_3_CN for **a3** and **b3**, respectively. Since a single ZnP covers ≈7 units of a single face of Ti_3_C_2_ or Ti_3_CN, we can estimate a total surface coverage of 8.4% and 10.9% for **a3** and **b3**, in the same order.

**Figure 4:**
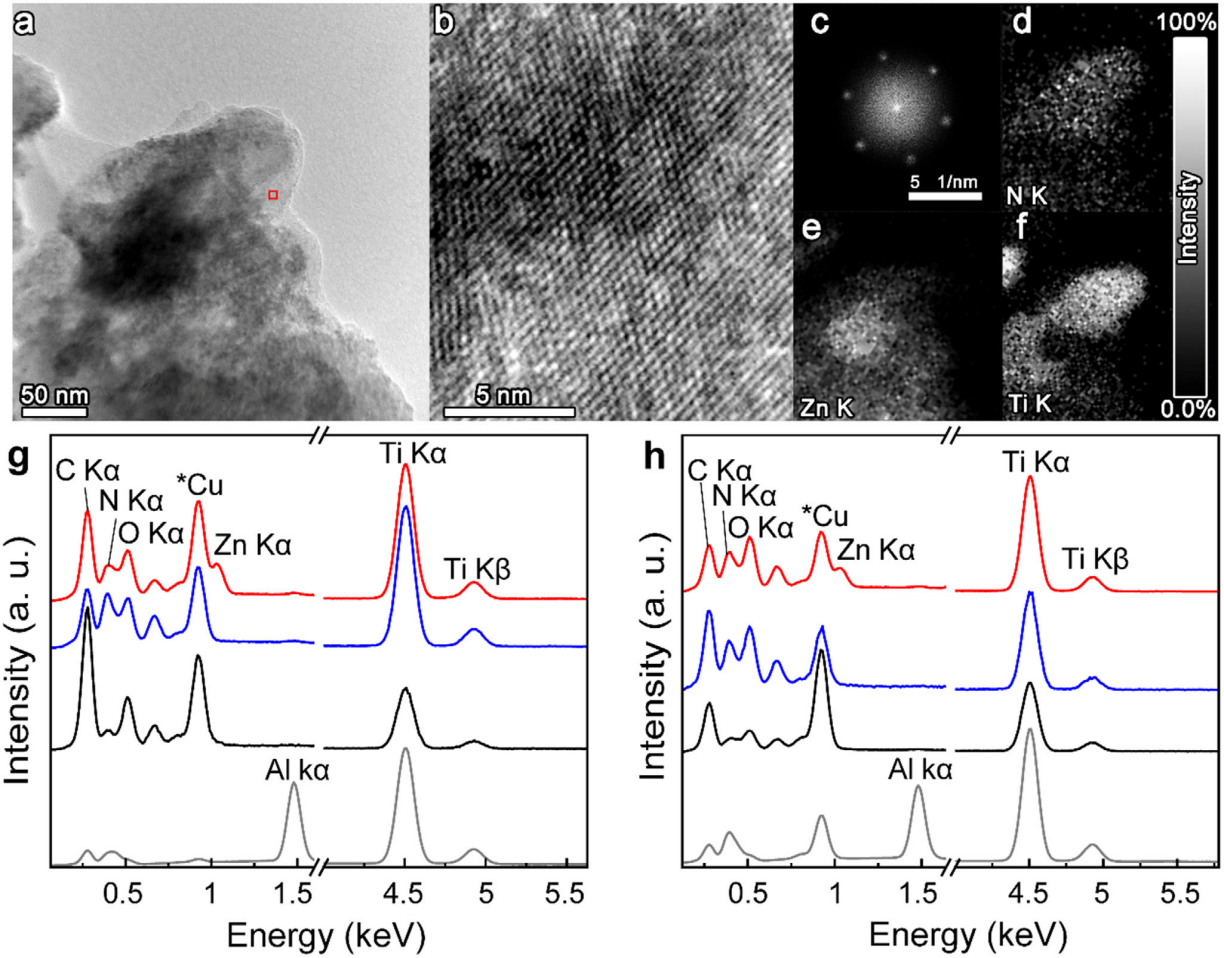
a,b) Low and high magnification TEM images of a hybrid material **a3**. c) FFT pattern of a hybrid material **a3**. d–f) EDS mappings of N, Zn, and Ti core levels in the TEM image (a). g) EDS spectra of Ti_3_AlC_2_ (gray), **a1** (black), **a2** (blue), **a3** (red). h) EDS spectra of Ti_3_AlCN (gray), **b1** (black), **b2** (blue), and **b3** (red).

The morphologies of the hybrid materials **a3** and **b3** were examined using TEM equipped with energy‐dispersive spectroscopy (EDS), as shown in **Figures** **4**a–f and S4 (Supporting Information), respectively. Both hybrid materials exhibited semitransparent areas, suggesting the presence of aggregated single‐ or few‐layered structures (Figure 4a; Figure S4a, Supporting Information). High‐magnification images (Figure 4b; Figure S4b, Supporting Information) revealed a lattice structure resembling graphene, primarily composed of three stacked Ti planes—the heaviest element present—while N and C planes were essentially invisible. The exposed Ti surface showed no holes, ensuring a high‐quality material. Fast Fourier Transform (FFT) analysis revealed a hexagonal pattern consistent with a graphene‐like lattice, characterized by a well‐compact, zero‐degree layered structure or a single layer (Figure 4c) and showing a Ti‐Ti distances of 2.7 Å. Notably, stains with a few nanometers square distributed around the sample were observed. These might correspond to areas enriched with amines or porphyrin,^[^ ^65^ ^]^ a hypothesis further supported by the EDS mappings. The EDS mappings confirmed a relatively uniform distribution of elements such as Ti, O, and Zn throughout the lattice of hybrid materials **a3** and **b3** (Figure 4d–f; Figure S4c–e, Supporting Information).

SEM was further employed to analyze the morphologies of **a1**, **a2**, **a3** and **b1**, **b2**, **b3** (Figures S5 and S6, Supporting Information). The MAX phases exhibited a uniform surface, whereas stacked layers became distinguishable after the etching process in **a1** and **b1**, forming the typical accordion‐like structure characteristic of MXenes. Additionally, all the materials (**a1**, **a2**, **a3,** and **b1**, **b2**, **b3**) showed a range of sizes, from 0.1 to 10 µm in length, with varying numbers of layers, from a few to multiple. Focusing on the most relevant changes in the EDS spectral mappings, the intensities of the Al Kα peak in Ti_3_AlC_2_ and Ti_3_AlCN clearly decreased in **a1** and **b1** (Figures S5e,f and S6e,f, Supporting Information). Spectral mappings of **a2** and **b2** revealed the presence of N Kα, while those of hybrid materials **a3** and **b3** showed the Zn Kα peak, confirming the high N and Zn content throughout the flakes (Figures S5g,h and Figure S6g,h, Supporting Information). The EDS spectra (Figure 4g,h) further support these observations. Specifically, Ti_3_AlC_2_ and Ti_3_AlCN exhibited an Al Kα peak at 1.48 keV, which was completely absent in **a1** and **b1**, while the Ti Kα peak at 4.50 keV remained. Light atoms, such as C Kα and N Kα, which contain peaks at 0.28 and 0.39 keV, respectively, are usually measured with low precision by EDX. Keeping this limitation in mind, it is still valuable to observe the changes, as they can provide qualitative information about each reaction carried out in the MXenes. For pristine materials, their signatures are relatively small because the titanium plane shields the inner layers. Nevertheless, both components are detected in **a1** and **b1**, indicating the presence of amines on the surface, which become more pronounced in **a2** and **b2**. Additionally, hybrid materials **a3** and **b3** exhibited a new Zn Kα peak at 1.03 keV, accompanied by increased intensities of the C Kα and O Kα signatures, confirming the presence of porphyrin. These results demonstrate that amines in **a2** and **b2** and Zn‐porphyrin in **a3** and **b3** are randomly distributed across the entire surface of Ti_3_C_2_ and Ti_3_CN, respectively. Finally, atomic force microscopy (AFM) analysis of isolated layers of **a3** revealed a predominant population of layers with a thickness of 1.5 nm, while those exceeding 5 nm were in the minority. Since a single layer of Ti_3_C_2_ functionalized on both sides is expected to have a thickness of 1.5 nm, this confirms the presence of a large population of single layers (see Figure S5i–l, Supporting Information).

### 2.2 Optical and Electronic Properties

At this stage, we can confidently confirm that the hybrid materials are based on MXenes covalently functionalized with porphyrin, ensuring significant preservation of the basal plane while achieving substantial porphyrin loading. ZnP is a photoactive molecule characterized by strong absorption and high quantum yield efficiency,^[^ ^36^, ^66^ ^]^ commonly transferring excitonic species such as electrons to 2D materials like graphene,^[^ ^22^ ^]^ thereby enhancing their optical performance. These studies are typically performed in liquid media, where a covalent bond is crucial to ensure stable contact between the two species while minimizing ZnP *π–π* interactions or aggregation. Therefore, our next goal is to carefully analyze the optical and electronic properties of the hybrid materials to understand the orbital interactions between the species and how the system responds to light stimuli.

**Figure 5:**
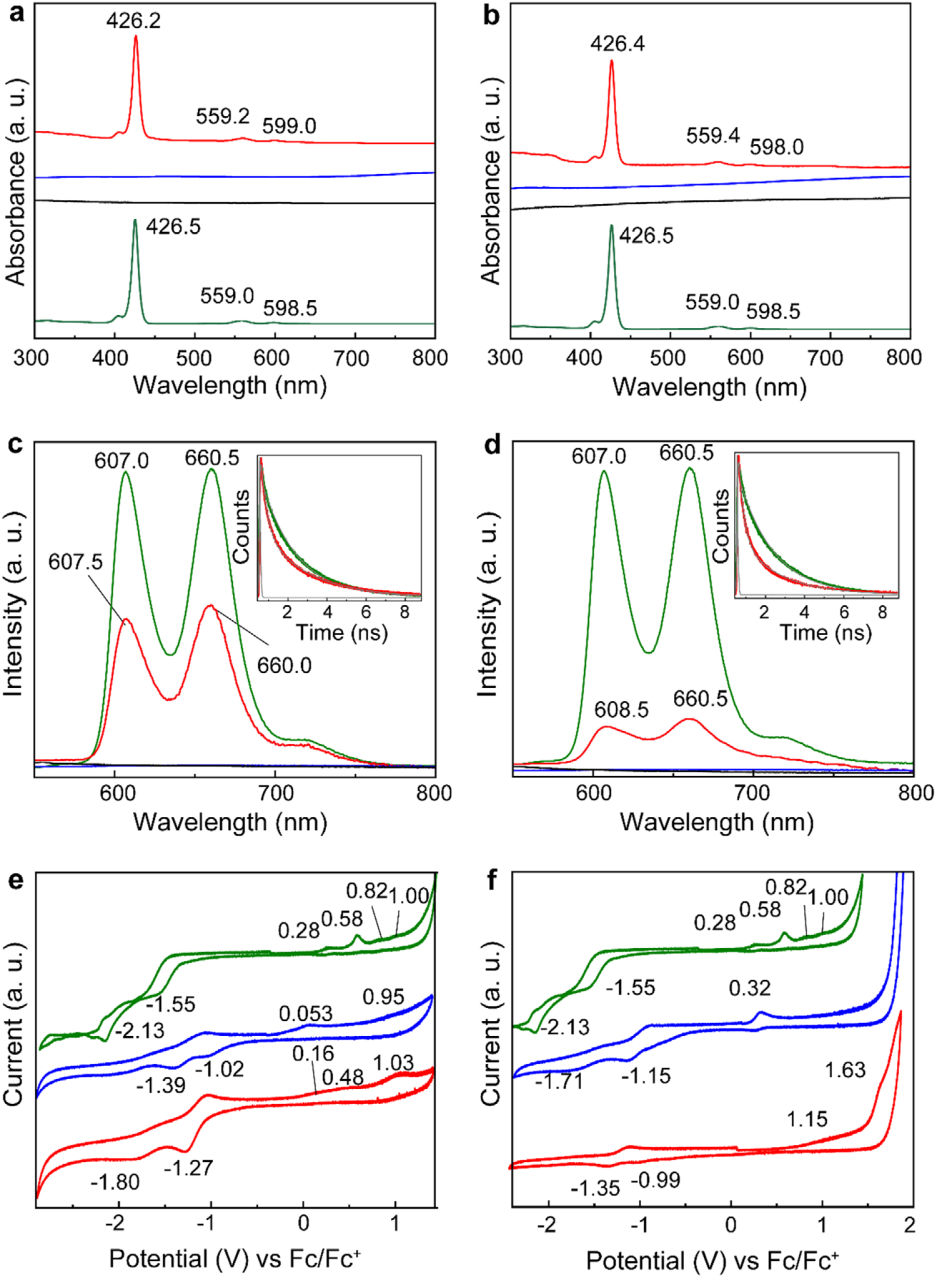
UV‒vis spectra a) ZnP (green), **a1** (black), **a2** (blue), **a3** (red), and b) ZnP (green), **b1** (black), **b2** (blue), **b3** (red), and PL emission spectra (exc. 420 nm) of c) ZnP (green), **a1** (black), **a2** (blue) and **a3** (red), and d) ZnP (green), **b1** (black), **b2** (blue), **b3** (red) in dimethylacetamide. Lifetimes of (Inset c) ZnP (green), **a3** (red), and IRF (gray), and (Inset d) ZnP (green), **a3** (red), and IRF (gray). Cyclic voltammetry of e) ZnP (green), **a2** (blue) and **a3** (red), and f) ZnP (green), **b2** (blue) and **b3** (red). CV was performed in acetonitrile using tetrabutylammonium hexafluorophosphate (TBAPF_6_) 0.1 m as the electrolyte. The working, reference, and counter electrodes were glassy carbon, Pt‐mesh, and Pt‐wire, respectively, with a scan rate of 25 mV s^‒1^.

**Figure** **5**a,b displays the UV‒vis absorption spectra of the hybrid materials **a3** and **b3**. ZnP exhibited three peaks at 426.4, 558.2, and 599.8 nm, corresponding to the Soret band and two Q‐bands of ZnP, respectively. In contrast, **a2** and **b2** show consistent absorption across the full spectrum. The spectra of **a3** and **b3** represent a superposition of both, with negligible changes in intensity or peak shifts.

PL emission under equal optical concentrations (Figure 5c,d), meaning the same amount of excitonic species, showed distinctive peaks at 607.0 and 660.5 nm for ZnP. These peaks are significantly quenched in **a3** and **b3**, and variations in the spectral shape suggest that excitonic species may be transferred from ZnP to Ti_3_C_2_ or Ti_3_CN. Moreover, the fluorescence quantum yield (φ^f^) of free ZnP, initially 3.1%, decreased to 1.3% and 1.2% in **a3** and **b3**, respectively. In short, the φ^f^ values were determined by comparing the linear slope of absorbance versus PL at different concentrations.

To fully prove that PL quenching is related to the direct interaction with MXenes, fluorescence lifetime experiments were performed. Pristine ZnP exhibits a monoexponential decay with a lifetime of 1.75 ns, whereas materials **a3** and **b3** show a faster decay, better fitting a biexponential model, with fast decay components of 0.30 and 0.26 ns, respectively (see inset in Figure S5c,d, Supporting Information). By employing Equations (1 and 2), we evaluated the quenching rate constant $K_{q}^{s}$ and the quantum yield *q_s_* of the singlet excited state, obtaining values of 2.76 and 3.27 ns^−1^ and 83% and 85%, respectively. Therefore, these data confirm the photogenerated charge transfer from ZnP to MXenes.

(1) $$K_{q}^{s}=\frac{1}{T_{f}}-\frac{1}{T_{o}}$$

(2) $$q_{s}\left(\%\right)=100\times K_{q}^{s}\times T_{f}$$

where *T_f_* and *T_o_* refer, in the same order, to the lifetime of the fast‐decaying in **a3** or **b3** and free ZnP components.

Cyclic voltammograms (CV) for **a1**, **a2**, **a3** and **b1**, **b2**, **b3** are shown in Figure 5e,f. The CV data provides valuable insights into the oxidation and reduction potentials of the materials, identifying the regions where holes or electrons can be accommodated, respectively. All oxidative and reductive potentials are reported versus ferrocene /ferrocenium (Fc/Fc^+^ = 0.0 V) redox couple and are summarized in Table S1 (Supporting Information). It is well established that the first oxidative and reductive potentials are directly correlated with the positions of the HOMO and LUMO, respectively, allowing a straightforward conversion from volts to electron volts (eV), since the Fermi level of Fc/Fc^+^ corresponds to ‒4.8 eV on the vacuum energy scale (Equations S2 and S3, Supporting Information). For free ZnP, the first oxidative and reductive potentials were 0.58 and ‒1.55 V, respectively, corresponding to a bandgap (BG) of 2.13 eV. This BG value aligns closely with the PL emission of porphyrin at 607 nm (2.05 eV). Additionally, applying the second reductive potential gives an energy of 2.95 eV, which is related to the absorption of the Soret band of porphyrin at 426 nm (2.92 eV), consistent with reports in the literature.^[^ ^66^ ^]^ Ti_3_C_2_‐based materials, such as **a2** and **a3**, were metallic, but the addition of functionalities, such as nitrogen derivatives, disrupts these properties, resulting in the opening of a bandgap.^[^ ^67^ ^]^ This behavior has been widely reported for other 2D materials, including graphene,^[^ ^68^ ^]^ MoS_2_,^[^ ^69^ ^]^ WS_2_,^[^ ^70^ ^]^ MoSe_2_,^[^ ^71^ ^]^ and MXenes,^[^ ^72^, ^73^ ^]^ such as other Ti_3_C_2_ systems.^[^ ^74^ ^]^ For **a2**, the first oxidation and reduction potentials were located at 0.05 and ‒1.02 V, respectively, corresponding to the conduction band minimum (CBM) and valence band maximum (VBM), yielding a BG of 1.07 eV. Ti_3_CN has an indirect bandgap with a reported BG value of 1.16 eV,^[^ ^7^ ^]^ which can also be tailored by the T*_x_* phase. **b2**, with an increased nitrogen content, exhibited slightly higher oxidation and reduction potentials at 0.32 and ‒1.15 V, respectively, yielding a BG of 1.43 eV. The cyclic voltammetry of **a3** and **b3** does not reflect a simple superposition of the ZnP and MXene signatures, likely due to strong orbital overlap between the ZnP and MXene. In fact, the ZnP signals appear smooth and are difficult to distinguish. However, the absence of significant shifts in the UV‒vis or PL spectra suggests minimal energetic variation in the ZnP orbitals. For instance, the only discernible peak of ZnP in **a3** was located at 0.48 eV, which is 0.1 V lower than that of free porphyrin and within the margin of error (± 0.2 V). Regarding the nanosheets, the variations observed in **a3** and **b3**, compared to **a2** and **b2**, suggest an orbital rearrangement in the MXenes, primarily lowering the ground states. While **a3** retains a narrow bandgap of 1.43 eV, the BG of **b3** expands to 2.14 eV. A vacuum energy diagram can be constructed, positioning the HOMO and LUMO of ZnP as well as the CBM and VBM of Ti_3_C_2_ and Ti_3_CN within **a3** and **b3** (Figure S7, Supporting Information).

Additionally, films of **a3** and **b3** were deposited on a gold substrate and analyzed using ultraviolet photoelectron spectroscopy (UPS) and low‐energy inverse photoelectron spectroscopy (LEIPS), which further confirmed the positions of the CBM and VBM for Ti_3_C_2_ and Ti_3_CN (Figure S8, Supporting Information). When comparing UPS and LEIPS values by employing Equations S4 and S5 (Supporting Information) with the oxidative and reductive potentials obtained by CV, no shifts greater than 0.23 eV were observed (Figure S7, Supporting Information). Indeed, the agreement between these techniques increases the reliability of our findings. The orbital diagrams suggest a type‐I alignment for the hybrid material **a3**. For **b3**, a type‐II alignment was initially observed, but the presence of trap states results in type‐I alignment behavior instead. In more detail, trap states originating from the pristine Ti_3_CN are expected to spread across the energy levels, accommodating holes or electrons depending on whether they lie above or below the Fermi level, respectively. The UPS binding energy diagram displays a small but constant current up to the Fermi level, a trend also reflected in the valence band energies and CV. Additionally, TEM images (Figure 4b) highlight nanometric regions with varied functionalization, suggesting the presence of areas that may still exhibit characteristics of the pristine material. These regions are likely responsible for creating a few, but significant trap states within the material. This analysis provides a critical foundation for interpreting the results discussed in the subsequent spectroelectrochemical (SEC) experiments.

**Figure 6:**
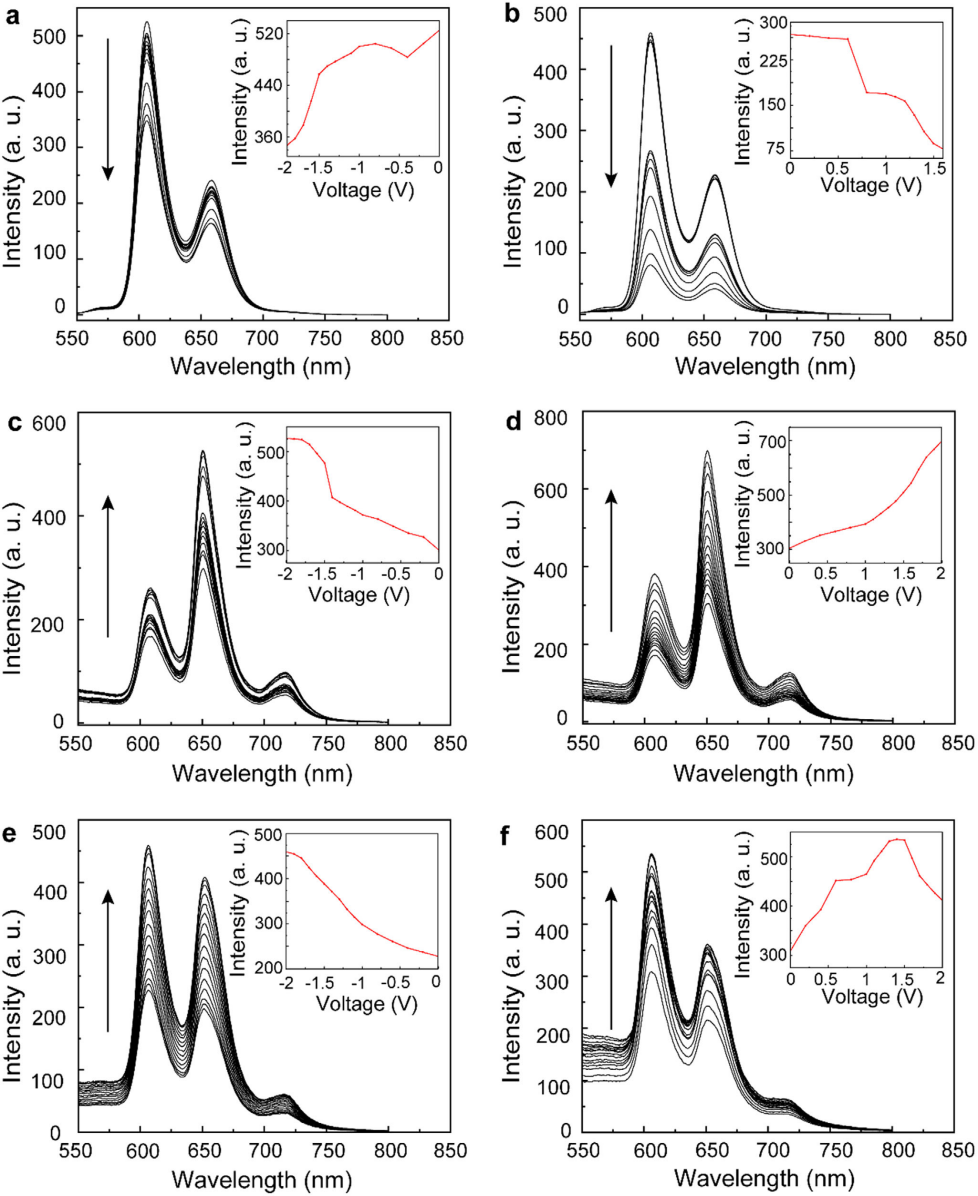
PL emission intensity under crescent voltages from 0 to 2 V for a) free ZnP, c) **a3**, and e) **b3**, and decrescent from 0 to ‒2 V for b) free ZnP, d) **a3,** and f) **b3**, in DMAc by employing 0.1 m of TBAPF_6_.

**Figure 7:**
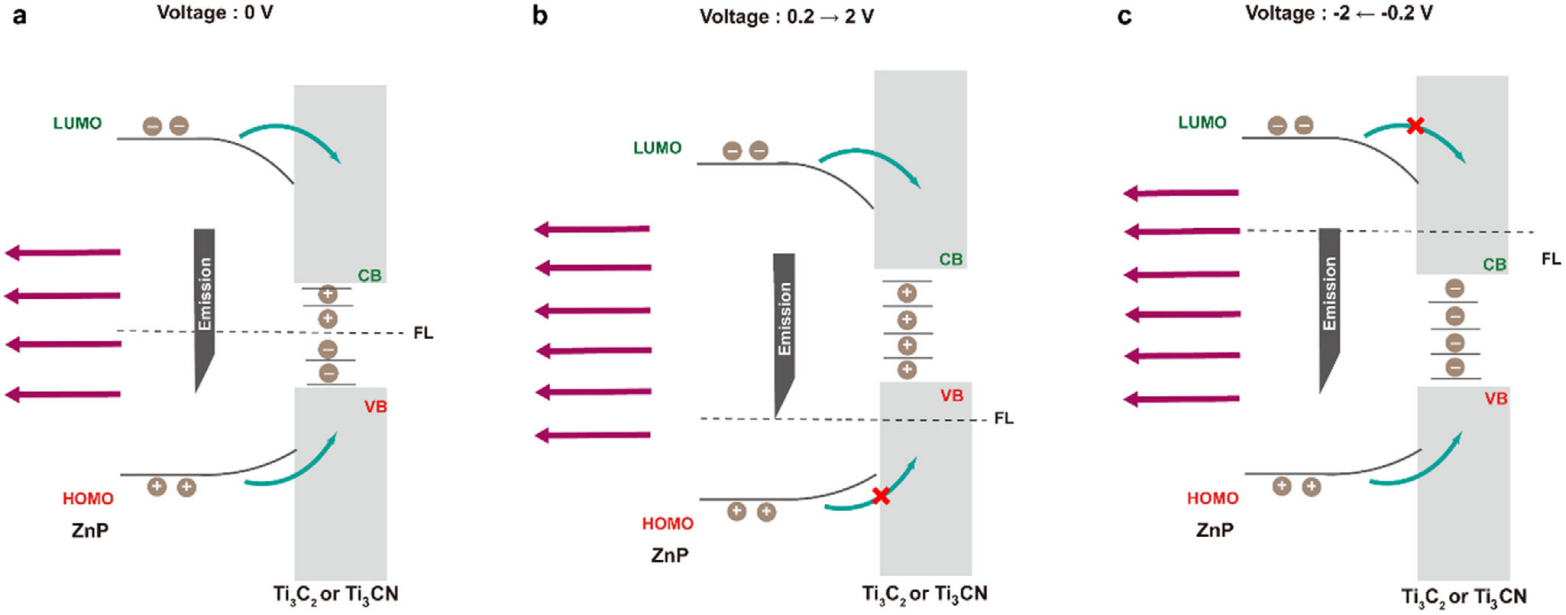
Carrier transfer process from ZnP to **a3** or **b3** under a) 0 V, b) positive, and c) negative voltage.

SEC assays were performed to further confirm the effect of light on the electronic states of ZnP, Ti_3_C_2_, and Ti_3_CN. The absorption spectra of free porphyrin exhibit a similar trend under different potentials when compared to **a3** and **b3**, suggesting limited interaction between the different species (Figure S9, Supporting Information). Interestingly, the PL emission of free porphyrin decreases under positive or negative voltage, whereas it increases in **a3** and **b3** (**Figure** **6**). In the absence of applied potential, the photogenerated holes and electrons of free porphyrin recombine effectively, without significant interactions with external species, resulting in strong PL emission (Figure S10a, Supporting Information). For hybrid materials **a3** and **b3**, the photoexcited electrons in the LUMO of porphyrin can transfer to the CBM of MXenes, while the photogenerated holes in the HOMO of porphyrin transfer to the VBM of MXenes, consistent with a type‐I alignment in both materials (**Figure** **7**a).

Under positive voltage, the Fermi level of the system is lowered, quenching the PL emission of free ZnP by transferring electrons to the surrounding environment (Figure S10b, Supporting Information). In the hybrid materials, lowering the Fermi level depletes electrons associated with the VBM and/or trap states of MXenes in **a3** and **b3**, resulting in a reduction of hole‐electron recombination events between the HOMO of ZnP and Ti_3_C_2_ or Ti_3_CN (Figure 7b). Conversely, under negative voltages, the Fermi level is elevated. For free ZnP, this leads to PL quenching due to hole transfer to the environment (Figure S10c, Supporting Information). However, in **a3** and **b3**, the trap states and CBM of Ti_3_C_2_ or Ti_3_CN become enriched with electrons, preventing recombination of the electrons in the LUMO of porphyrin with the excited states of MXenes (Figure 7c). It should be mentioned that the voltage‐dependent behavior observed in free porphyrin also influences ZnP within **a3** and **b3**. However, the interactions between MXenes and ZnP predominate due to the covalent linkage, ensuring consistent and close interaction between the two species. In summary, UV‒vis, PL, CV, UPS, and LEIPS provide in‐depth information about the band structure of these materials, suggesting a type‐I behavior alignment in both **a3** and **b3**. While direct evidence of energy transfer between ZnP and MXenes remains inconclusive, the SEC experiments confirm electron and hole transfer from porphyrin to Ti_3_C_2_ and Ti_3_CN within **a3** and **b3**.

### 2.3 Photo‐Device Applications

So far, we have demonstrated the covalent functionalization reaction between ZnP and MXenes in a two‐step process. This functionalization occurs on the basal plane, layer‐by‐layer, enhancing the interaction between both components. We then investigated the optical and electronic interaction of ZnP with Ti_3_C_2_ and Ti_3_CN, which results in a type‐I band alignment. In this configuration, ZnP facilitates the transfer of holes and electrons, thereby enhancing the carriers of the hybrid materials. The next logical step is to explore an application where the new properties of **a3** and **b3** materials are relevant. To this end, we can develop photosensors based on ultrathin layers and analyze the photo‐response (Equation S6, Supporting Information), responsivity (Equation S7, Supporting Information), external quantum efficiency (Equation S8, Supporting Information), specific detectivity (Equation S9, Supporting Information), and superficial conductivity (Equation S10, Supporting Information).

**Figure 8:**
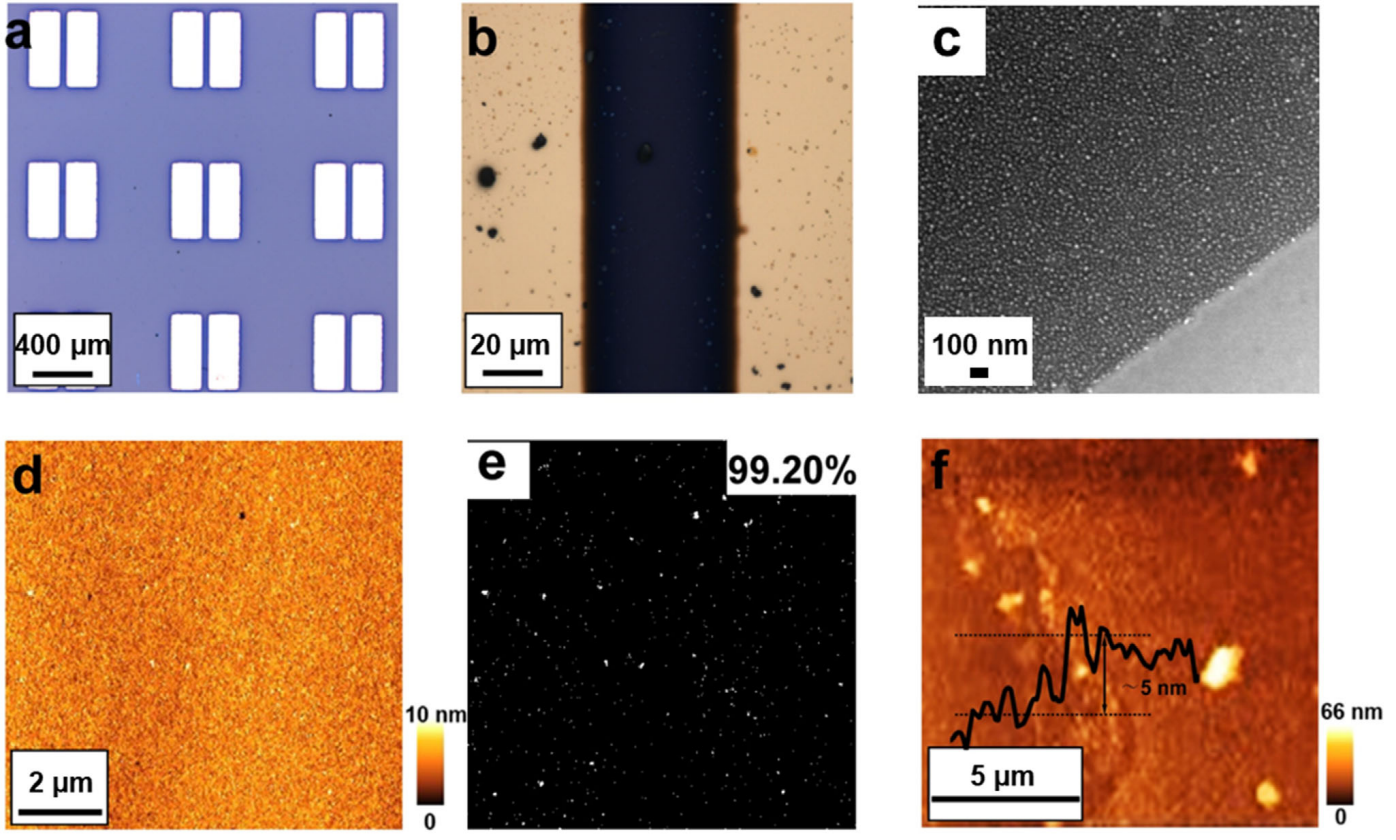
a) Optical microscope image of electrodes employed for **a2**, **b2**, **a3,** and **b3**. b) Optical microscope showing the channel of **a3** or **b3**‐based nanodevices. c) SEM of a scratched area for evaluating the quality of a representative film for **a3** or **b3**. d) AFM of a representative film for **a3** or **b3**. e) Nanofilm mapping coverage evaluated from Figure 8d, and calculated by Image J. f) AFM of a scratched area for evaluating the thickness of a representative film for **a3** or **b3**.

Regarding device fabrication, the spontaneous assembly technique^[^ ^75^ ^]^ outperforms Langmuir‐Blodgett^[^ ^76^ ^]^ or single‐droplet.^[^ ^77^ ^]^ The optimal conditions are described in the Supporting Information. It should be noted that when films based on pristine materials (**a2** or **b2**) undergo functionalization, only the first layer of the nanofilm can be decorated with porphyrin, leaving the inner layers unaffected. This limitation compromises one of the key advantages of covalent functionalization, namely, layer‐by‐layer functionalization. Therefore, the devices were constructed directly with **a3** and **b3**. On the other hand, an accurate comparison between pristine and functionalized materials requires evaluating the same device before and after functionalization, which is not feasible in this study. To address this limitation, several devices of 50 pairs of electrodes, each with the same dimensions (50 µm channel width and 100 µm length), were fabricated for **a2**, **b2**, **a3**, and **b3**. All devices exhibited similar thicknesses, proximately 5 nm, and comparable quality, with 99.20% surface coverage, as evaluated using optical microscopy, AFM, and SEM (**Figure** **8**). Devices constructed with **a3** achieved dark currents of 1.7 µA (8.4 nS sq^‒1^), while those with **b3** reached 9.0 µA (45 nS sq^‒1^). In contrast, devices based on **a2** and **b2** demonstrated consistently poor performance, with currents in the fA range, as illustrated in Figure S11 (Supporting Information).

**Figure 9:**
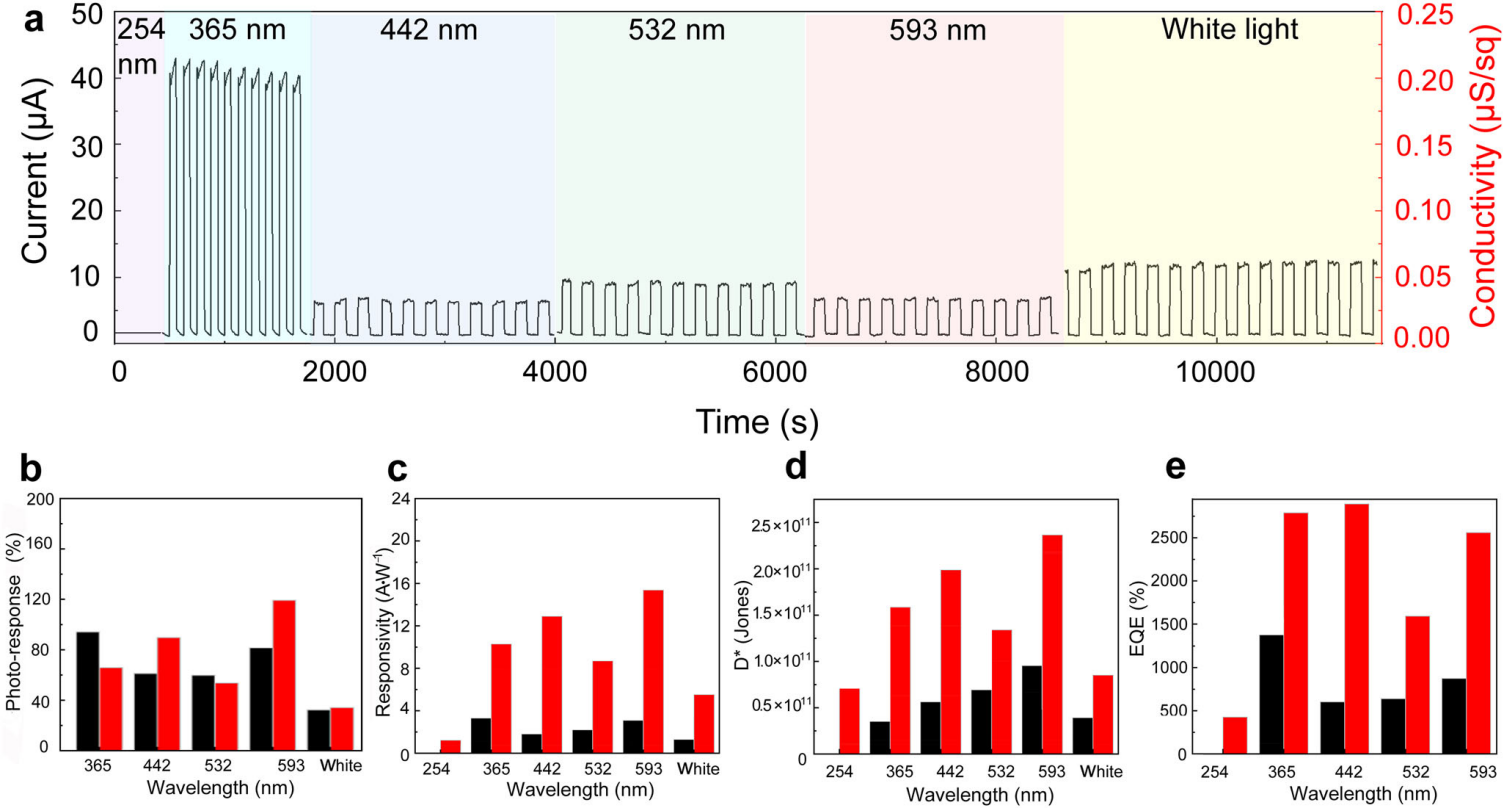
a) Performance of **a3** devices under different light‐wavelength irradiation, b) photo‐response at 1 mW cm^−2^, c) responsivity, d) specific detectivity, and e) EQE of **a3** (black) and **b3** (red) based devices under different wavelength excitation and bias voltage of 5 V.

Next, the optical activity of devices based on **a3** and **b3** was evaluated under various light sources, including UV‐B (254 nm; 4.8 eV), UV‐A (365 nm; 3.41 eV), blue (442 nm; 2.81 eV), green (532 nm; 2.34 eV), red (593 nm; 2.10 eV), and white light, with incident powers of 0.9, 40, 10, 13, 6 and 31 mW cm^‒2^, respectively. While pronounced photo‐responses were observed from UV‐A to red wavelengths for **a3** and **b3** (**Figure** **9**a; Figure S12, Supporting Information), **a2** and **b2** showed no optical performance (Figure S13, Supporting Information). Moreover, hybrid materials **a3** and **b3** demonstrated minimal drift over several light pulses (3 min), ensuring high system stability for extended periods up to 4 h. Although instrument limitations prevented precise measurement, the photo‐response times and recovery times were estimated to be below 1 s. As illustrated in Figure 9a, the robust photo‐response, with on‐off ratios superior to 25‐fold, highlights the potential of these materials as efficient photosensors. For comparison purposes, Figure 9b shows the normalized photoresponse against the incident light power, clearly demonstrating enhanced performance in the visible region.

Further insights were obtained through responsivity and specific detectivity measurements (Figure 9c,d). Porphyrin‐based MXenes exhibited a significant improvement in the visible region compared to pristine materials (Figure 9; Figures S12 and S13, Supporting Information). The responsivity and specific detectivity values for **a3** and **b3** ranged from 1.40 to 15.0 A W^‒1^ and 3.0–23.0 × 10^10^ Jones under any wavelength within the visible range. Material **a3** exhibited equal responsivity and specific detectivity under UV‐A and red light, with values of 3.50 A W^‒1^ and 9.4 × 10^10^ Jones, respectively, which are close to those for green and blue light. For material **b3**, red light showed the best performance, reaching values of 15.0 A W^‒1^ and 23.0 × 10^10^ Jones. Notably, **b3** under blue and red irradiation surpassed the responsivity and specific detectivity values of UV‐A by ≈20% and 30%, respectively. The strong light response observed in our instrument may result from a combination of factors, making it difficult to pinpoint a single cause. Nonetheless, we have analyzed the data and propose a plausible hypothesis, which should be considered as an interpretation rather than a conclusive fact. Focusing on hybrid material **b3**, the highest responsivity was observed under red light (2.10 eV), close to the Ti_3_CN bandgap within **b3**, previously calculated as 2.14 eV, likely enhanced by the Q band of porphyrin at 599 nm (2.08 eV). The second‐highest responsivity occurred in the blue region, which aligns with the absorption of the Soret band of porphyrin, followed by UV‐A (3.41 eV), potentially attributed to a direct transition from the VBM of Ti_3_CN to the LUMO^+1^ of porphyrin, calculated at 3.28 eV. The response at 2.34 eV in the green region might be influenced by the Q band absorption at 558 nm (2.23 eV). In contrast, the lack of sensitivity to UV‐B is likely due to its high energy, which does not correspond to any plausible transitions in the system. Although additional mechanisms may contribute, it is clear that porphyrin plays a crucial role in enhancing the photoactivity of these hybrid materials.

The quantum efficiency of **a3** and **b3** (Figure 9e) is also an important parameter in photosensors, with values of 1300% and 2830% under a bias voltage of 5 V, which is approximately one order of magnitude larger than the average of previous studies on MXene‐based materials.^[^ ^78^ ^]^ This indicates that each incident photon on the hybrid materials **a3** and **b3** is capable of generating multiple charge carriers (holes and electrons), highlighting the high performance of the device. Moreover, the elevated quantum efficiency and responsivity indicate that electrons and holes transferred from porphyrin to MXenes are not self‐annihilated but instead disperse around the basal plane, contributing as excitonic carriers.

**Figure 10:**
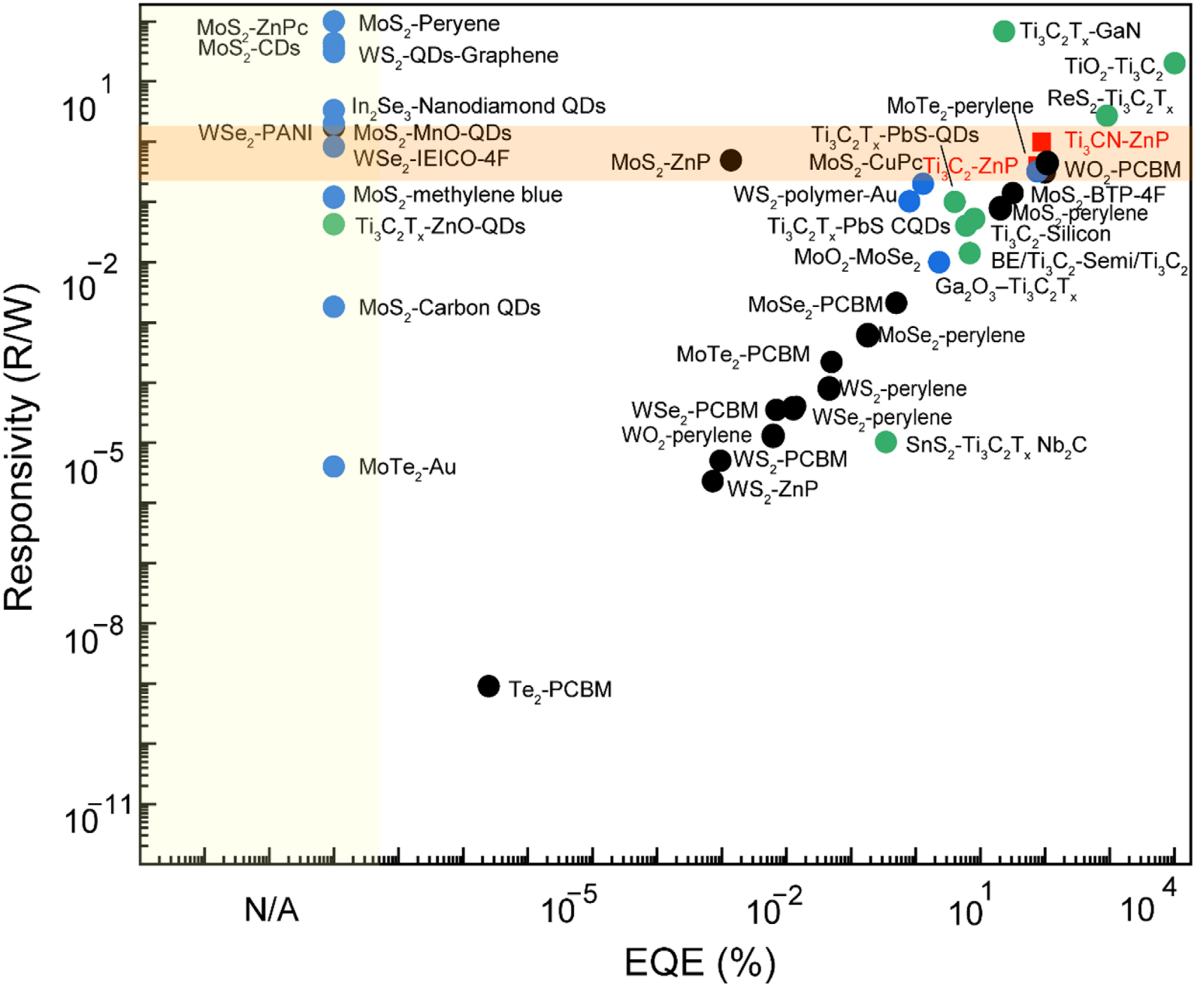
Comparison of the responsivity and EQE^[^ ^35^, ^66^, ^78^, ^79^, ^80^, ^81^, ^82^, ^83^, ^84^, ^85^, ^86^, ^87^, ^88^, ^89^, ^90^, ^91^, ^92^, ^93^, ^94^, ^95^, ^96^, ^97^, ^98^ ^]^ of **a3** and **b3** with other 2D hybrid materials. The yellow area represents studies with only responsivity data. The red rectangles represent work in this study. The black dots indicate covalent functionalization studies. The blue dots correspond to non‐covalent functionalization with organic molecules. The green dots represent studies related to MXenes.

Finally, we compared the best responsivity, quantum efficiency (**Figure** **10**), and photo‐response (Figure S14, Supporting Information) of **a3** or **b3** devices with those reported in the literature.^[^ ^35^, ^44^, ^66^, ^78^, ^79^, ^80^, ^81^, ^82^, ^83^, ^84^, ^85^, ^86^, ^87^, ^88^, ^89^, ^90^, ^91^, ^92^, ^93^, ^94^, ^95^, ^96^, ^97^, ^98^ ^]^ Among MXene‐based materials, **a3** or **b3** devices occupy a privileged position, surpassed only by TiO_2_/MXene^[^ ^35^ ^]^ and ReS_2_‐Ti_3_C_2_.^[^ ^82^ ^]^ Most functionalized Ti_3_C_2_ or Ti_3_CN focus on UV‐A and UV‐B regions, leaving the visible range either unexplored or inactive, with rejection ratios exceeding three orders of magnitude.^[^ ^78^ ^]^ In this context, the performance of **a3** and **b3** remains peerless. We then evaluated our results against well‐established 2D material‐based photodetectors, particularly those targeting visible light, such as TMDs. Covalent functionalization techniques in this field are scarce, with only a few examples involving PCBM,^[^ ^79^ ^]^ porphyrin,^[^ ^99^, ^100^, ^101^, ^102^ ^]^ and pyrene.^[^ ^45^ ^]^ Our devices demonstrated superior performance in responsivity. In terms of dark‐to‐light current ratio (photo‐response), only PCBM‐MoS_2_ and MoS_2_‐ZnP outperform **a3** or **b3**. In a broader context, when including non‐covalent systems and inorganic species such as nanoparticles, **a3** or **b3** devices exhibit average performance in responsivity and EQE. Nevertheless, by covalently functionalizing optically inactive Ti_3_C_2_ and Ti_3_CN with porphyrins, we have developed highly effective photodetectors capable of covering the entire visible spectrum, rivaling the best results reported in the literature.

## 3 Conclusion

In conclusion, the T*_x_* phase of MXenes was modified to carry alkyl amines, enabling straightforward functionalization with porphyrin. The flexibility of the alkyl amines facilitates close electronic interaction between the aromatic porphyrin skeleton and the MXenes’ basal plane. This process allowed for large functionalization loading, as confirmed by the Kaiser test and TGA, with XPS and IR providing definitive evidence of covalent functionalization.

A type‐I band alignment was observed between ZnP and Ti_3_C_2_ or Ti_3_CN, while SEC experiments suggest efficient hole and electron transfer events under light irradiation, increasing the number of carriers on the surface of MXenes. Unlike other 2D materials, such as graphene, basal functionalization of MXenes does not introduce defects; instead, it stabilizes the Ti layers, enabling high‐quality nanodevices.^[^ ^20^ ^]^ Photodetectors based on these nanofilms showed significant improvements in current and photo‐response after functionalization, especially under visible light, demonstrating performance on par with the most advanced MXene‐based devices.^[^ ^20^, ^34^ ^]^ Specifically, the maximum values of responsivity and external quantum efficiency reached 10.35 A W^‒1^ and 2440%, respectively. This work highlights the potential of chromophore‐functionalized MXenes for light‐related nanodevice applications, distinguishing them from other 2D materials.

## 4 Experimental Section

### Synthesis of MXenes Nanosheets

In a 25 mL vial containing 5 mL of ethylenediamine (EDA), 3.7 g of ammonium fluoride (NH_4_F) was slowly added under stirring, forming a white solid as NH_3_ bubbles were released. Then, 5 mL of HCl (35%) was slowly added, followed by 500 mg of Ti_3_AlC_2_ or Ti_3_AlCN. The reaction was kept under stirring at 60 °C for Ti_3_AlC_2_ or at reflux temperature for Ti_3_AlCN under a nitrogen atmosphere. During the etching process, bubbles were observed due to the production of hydrogen gas. After stirring for 1 day with occasional sonication, the solution was filtered using PTFE membranes with a pore size of 0.2 µm and sequentially washed with water, N‐Methyl‐2‐Pyrrolidone (NMP), and methanol, resulting in a black solid.

Next, the solid was tip‐sonicated for 1 h in DMSO using a Branson Digital Sonifier SFX 550 set to 70% amplitude. The solid was obtained after filtering the original solutions of Ti_3_AlC_2_ or Ti_3_AlCN, respectively, using PTFE membranes with a pore size of 0.2 µm and sequentially washing with water, trimethylamine, N‐Methyl‐2‐Pyrrolidone (NMP), and methanol. The product was tip‐sonication for 1 h in ethylenediaminetetraacetic acid (EDTA) solution, which was prepared by dissolving EDTA in NaOH solution with pH = 12. The solid products **a1** and **b1** were obtained after the same filtration washing process.

The materials **a1** or **b1** were immersed in 20 mL of EDA for 1 day at 70 °C for **a1** or 100 °C for **b1** under a nitrogen atmosphere. The products **a2** and **b2** were obtained after filtration by employing PTFE membranes with a pore size of 0.2 µm and washing with water and acetone.

### Synthesis of ZnP‐MXenes **a3** and **b3**

A mixture of 20 mg DCC, 20 mg DMAP, 10 mg ZnP (which was 5‐(4‐carboxyphenyl)‐10,15,20‐(triphenyl) porphyrin Zn^2+^), and 20 mg of **a2** or **b2** was dispersed in 10 mL of dry pyridine. The reaction mixture was stirred at 40 °C for 5 days with occasional sonication, while small amounts of DCC were added periodically. After this period, the reaction mixture was filtered using PTFE membranes with a pore size of 0.2 µm and washed with 30 mL of water and 30 mL of acetone. The hybrid materials **a3** and **b3** were then collected as a solid.

### Fabrication of Photodevice

A 1 × 1 cm^2^ SiO_2_/Si (90 nm) substrate was cleaned with acetone and covered with a metal mask featuring 50 pairs of electrodes with channel areas of 500 × 50 µm^2^ square hollow pattern. Ti/Au electrodes (5/50 nm) were deposited on the surface using electron beam evaporation. Next, 250 µL of a solution of **a2**, **a3,** or **b2**, **b3** in DMSO (4 g L^‒1^) was added to a 40 mL vessel containing the vertically placed substrate, deep in ≈30 mL of water. Then, 7 mL of a mixture of ethanol and water (v/v = 1:1.5) was carefully dropped onto the water's surface. After several seconds, a nanofilm based on **a2**, **a3** or **b2**, **b3** was formed at the water interface and deposited onto the substrate by removing the water.

## Conflict of Interest

The authors declare no conflict of interest.

## Supporting information



Supporting Information

## Data Availability

The data that support the findings of this study are available in the supplementary material of this article.
