# Supplementary material for: Covalent Functionalization of MXenes with Porphyrin for Visible‐Light Activation in Energy Conversion Nanodevices
Source: Small. 2025 Aug 3;21(38):e03895. doi: 10.1002/smll.202503895 (PMC12462596; doi:10.1002/smll.202503895)
Supplement: Supplementary file 1 — Supporting Information [file SMLL-21-e03895-s001.docx]

Supporting information

Covalent Functionalization of MXenes with Porphyrin for Visible-Light Activation in Energy Conversion Nanodevices

Hong Li,^‡^ Ruben Canton-Vitoria,^‡*^ Yuto Urano, Sudhanshu Kumar Nayak, Eisuke Yamamoto, Makoto Kobayashi, Ryo Kitaura, Minoru Osada^*^

**CHEMICALS**

Ethylenediamine (Purity ~99 %), NH_4_F(97 %), HCl (35 %), methanol (99.8 %), Dimethyl sulfoxide (>99.0 %) and N-Methyl-2-pyrrolidone (>99.0 %) were purchased from KISHIDA Chemical Co., Ltd. N,N-Dimethylformamide, super dehydrated (Purity > 99.5 %), N,N-Dimethylacetamide (DMAs); (99.95 %), super dehydrated (Purity > 99.5 %) and triethylamine (>99.0 %) were purchased from FUJIFILM Wako Chemicals U.S.A. Co., Ltd. MAX phase material (Ti_3_AlC_2_ (>=90 %), Ti_3_AlCN (>=90 %), N,N'-Dicyclohexylcarbodiimide (>=99 %) were purchased from Sigma Aldrich Co., Ltd. 4-Dimethylaminopyridine (>99.0 %) were purchased from TCL Co., Ltd.

**INSTRUMENTS**

TGA:

Thermal tests were performed using the STA7200 Thermal Analysis System from HITACHI. The hybrid materials solids (**a1**, **b1**, **a2**, **b2**, **a3** or **b3**) were obtained by filtration process. Typically, 2-3 mg of the sample was put on an alumina substrate and heated at a rate of 10 °C/min under 250 ccm of nitrogen flow.

XPS, UPS and LEIPS:

A drop of 4 mg·mL^–1^ of the target material in DMAc was deposited onto a silicon wafer which is covered by 50 nm of gold, and dry at 110 ^o^C. Subsequently, a PHI Versa Probe III instrument was utilized to record the spectrum. The pressure of the system was smaller than 1x10^–7^ Torr in any moment. For XPS, Ar gas was employed for charge removal. For UPS measurements, the first band of Helium (21.22 eV), stabilized at 0.59 kV and 100 mA, was used as a reference. A sample bias of –5 V was applied for kinetic energies. For LEIPS measurements, a neutralization gun with a current intensity of 1 μA and 20 V and a 4.77 band pass filter energy was employed. The position of the conduction band minimum (CBM) was obtained from the point where the LEIPS spectrum intersects the kinetic energy axis.

SEM:

The morphology of the samples was observed by FE-SEM (JSM-7610FPlus). Samples were measured in a SiO_2_ substrate or TEM grid.

TEM:

A JEM100Plus instrument equipped with EDX was employed for measuring the samples, at 200 KV, by employing TEM and/or STEM methodology. The copper mesh was immersed in the nanosheet solution to fish out the nanosheets. Part of nanosheets will attach on the surface of the copper mesh for TEM characterization.

RAMAN:

Materials **a1**, **b1**, **a2**, **b2**, **a3** or **b3** were examined as powders deposited on SiO_2_ substrate and characterized with a Horiba Jobin-Yvon LabRAM HR-800. Raman measurements were performed in air with a 532 nm laser intensity of less than 1 % of 80 mW·cm^–2^ with a resolution of 0.5 cm^–1^ and with magnifications of ×50 or ×10.

FT-IR:

Experiment was performed using the IR-4600 in air. Baseline was collected by 64 scans with a resolution of 2 cm^–1^ before each experiment. Around 1 mg of the samples (**a1**, **b1**, **a2**, **b2**, **a3** or **b3**) were ground mixed with KBr (80～90 mg) and pressed until a pellet approximately 1 mm thick was formed, which was then positioned in the IR beam.

UV-Vis:

UV-Visible Spectrometer V-770 was employed. A drop of a solution of **a1**, **b1**, **a2**, **b2**, **a3** or **b3** (4 g·mL^–1^) in DMAc were diluted with ~5 mL of DMAc, until optical concentration. The diluted solution is poured into a quartz cube container (1x1x4 cm^3^ cuvette) for characterization.

PL emission:

Jasco FP-8550 instrument was employed. A drop of 4 mg·mL^–1^ of the target material was diluted with ~5 mL of DMAc. The diluted solution is poured into a quartz cube container (1x1x4 cm^3^ cuvette) for characterization.

Lifetime.

The excitation wavelength was 450 nm and the PL emission was recorded between 500-900 nm. The laser employed was a Mai Tai Tunable femtosecond laser, 100 fs pulse duration, 80 MHz repetition rate, 300 sec, and 14 μW·cm^–2^ with a sample concentration of 0.2 mg·mL^–1^ for **a3** or **b3** in dimethyl sulfoxide.

Optical Microscope:

A film of the target materials was evaluated by a confocal laser microscopy (LEXT OLS4000) instrument.

AFM:

A film of the target materials in a SiO_2_/Si substrate was evaluated by instrument 5100N, at 0.5 Hz, and employing the tapping mode.

Cyclic voltammetry (CV):

The Potentiostat Electrochemistry System Keithley 2450-EC was used to perform the experiment. 2×2 cm^2^ Pt mesh served as the counter electrode, and a Pt wire was used as the reference electrode. A thin film of the analyzed material was deposited onto a 7 mm^2^ glass carbon electrode. Commercial tetrabutylammonium hexafluorophosphate (TBAPF_6_), recrystallized twice, was used as the electrolyte at a molar concentration of 0.1M. Commercial ultra-dry acetonitrile was employed as the solvent. Prior to starting the experiment, pure N_2_ was bubbled through the solution to completely remove oxygen species. Finally, commercial ferrocene purified by sublimation was employed to calibrate the data to 0.0 V.

Spectroelectrochemical experiments:

The previously described CV instrument was connected to the UV-vis or PL instrument, and a spectroelectrochemical (SEC) cuvette with dimensions of 0.2 × 1 × 0.5 cm^3^ was used. A mesh electrode with dimensions of 0.7 × 0.6 cm^2^, 80% transmittance, and a square pore size of 320 microns, interfaced with Pt wires of 60 microns, served as the working electrode. A platinum wire electrode was used as the counter electrode, while a gold electrode functioned as the reference electrode. PL and UV-vis data were recorded after a 30-second potential stabilization period. DMAc was selected as the solvent, TBAPF_6_ was used as the electrolyte. The experiment was conducted from 0 to 2 volts, and after refreshing the solution, measurements were recorded from 0 to -2 volts.

Semiconducting analyzer:

The optoelectronic devices were characterized by Semiconducting analyzer (Keithley 4200 SCS Agilent E4980A) under vacuum environment and room temperature. Firstly, the sample was placed on the metal chassis of the probe station. Then, we turned on the vacuum pump to create a vacuum environment and contact the probes with device electrodes. During the measurement, various light sources, including UV-B (254 nm; 4.8 eV), UV-A (365 nm; 3.41 eV), blue (442 nm; 2.81 eV), green (532 nm; 2.34 eV), red (593 nm; 2.10 eV), and white light, with incident powers of 0.9, 40, 10, 13, 6 and 31 mW·cm^‒2^, respectively, were used.

Metal deposition technique:

On clean Si/SiO_2_ (90 nm) substrate, 50 pairs of Ti/Au electrodes were fabricated by E-Beam deposition system (Sanyu SVC-700LEB), through modifying the values of E-Gun power supply to control thickness of metal (Titanium and gold), Ti/Au (5 nm/50 nm) electrodes with a channel width of 50 μm and a length of 500 μm were obtained.

Film fabrication:

In this approach, a substrate covered by electrodes was placed at the bottom of a vessel. Then, 250 µL of a solution of **a2**, **a3** or **b2**, **b3** in DMSO (4 g L^‒1^) was added to a 40 mL vessel containing the vertically placed substrate (, deep in around 30 mL of water. Next, 7 mL of a mixture of ethanol and water (v/v = 1:1.5) was carefully dropped onto the water’s surface. After several seconds, a nanofilm based of **a2**, **a3** or **b2**, **b3** was formed at the water interface and deposited onto the substrate by removing the water.

**Supplementary Note 1. Formula of TGA**

The degree of functionalization (from TGA curve) was calculated by employing formula Eq. S1:

((Equation)) (S1)

$$U_{o/I}=\frac{{O_{Mw}}/{M_{L}}}{{I_{Mw}}/{R_{m}}}$$

O_MW_ represents the molecular weight of the organic species (like EDA or porphyrin). I_MW_ represents the molecular weight of the inorganic species (Ti_3_CN or Ti_3_C_2_).M_L_ denotes the mass loss during thermal decomposition (expressed as a percentage). R_m_ denotes the residual mass during the thermal degradation (expressed as a percentage). U_O/I_ indicates the number of units of the inorganic species (based on its chemical composition, e.g., Ti_3_CN and Ti_3_C_2_) per every organic molecule. In this work, the mass loss between 150 and 500 °C was used, as it primarily corresponds to the degradation of the organic species without affecting the inorganic part.

**Supplementary Note 2. Formulas of CV**

Electrochemistry may be considered controversial for evaluating the positions of energy gaps in materials. The primary reason for this is that during oxidative or reductive processes, the material could undergo chemical changes that modify its electronic states. However, it is well known that ZnP and MXenes are chemically stable under oxidative or reductive potentials, during the experimental process, suggesting that the current methodology is applicable.

Cyclic voltammetry, therefore, might not be suitable for evaluating novel materials. However, since our system involves the interaction of two well-known and chemically stable materials (ZnP and MXenes), simple math calculations should suffice to verify whether the oxidative or reductive positions align with the values reported in the literature. This approach acknowledges the limitations of the system while providing reliable results. Additionally, the HOMO and LUMO gaps of perylene align well with its optical gap, LEIPS and UPS.

The HOMO or VB (from the oxidation potential) was calculated by employing formula Eq. S2:

((Equation)) (S2)

$$HOMO=-\left( 4.8-E_{Fc/{Fc}^{+}}{+E}_{ox}^{onset} \right)eV$$

$E_{ox}^{onset}$ is the onset oxidation potential measured (in volts) relative to Fc/Fc^+^. Since the signatures of Fc/Fc^+^ were shifted to 0.0 V, the value of $E_{Fc/{Fc}^{+}}$ is also 0.0 V.

LUMO or CB (from the reduction potential) was calculated by employing the Eq. S3:

((Equation)) (S3)

$$LUMO=-\left( 4.8-E_{Fc/{Fc}^{+}}+E_{red}^{onset} \right)eV$$

$E_{red}^{onset}$ is the onset reduction potential measured (in volts) relative to Fc/Fc^+^. Since the signatures of Fc/Fc^+^ were shifted to 0.0 V, the value of$E_{Fc/{Fc}^{+}}$is also 0.0 eV.

**Supplementary Note 2. Energy band structure**

According to the UPS spectra with –5 V bias, we can observe the secondary electron cutoff (SECO) and Fermi level (*E_f_*). The valence band maximum (*E_VBM_*) is defined as the following Eq. (S4):

((Equation)) (S4)

$$E_{VBM}=h\upsilon-(E_{SECO}-E_{f})$$

Where *hυ* is the work function of He (21.2 eV), and the *E_SECO_* is the energy of SECO. We obtained the *E_VBM_* of pristine **a3**, **b3** are –4.94 eV, –5.75 eV, respectively.

Furthermore, the conduction band minimum (*E_CBM_*) can be extracted from the Eq. (S5):

((Equation)) (S5)

$$E_{CBM}=E_{IF.LEIPS}-E_{onset}+{h\upsilon}_{BPF}$$

Where: *E_IF.LEIPS_* is the inflection points of LEET curves, the *E_onset_* are onset energy observed in the original LEIPS spectrum. *hυ_BPF_* is the energy of band pass filter (4.77 eV). Based on the Eq. (4), we adjusted the original LEIPS spectra and obtained the *E_CBM_* of **a3**, **b3** both are –3.88 eV.

**Supplementary Note 3. Photo-device analysis.**

In addition, the photo-response (R), responsivity (R_λ_), the external quantum efficiency (EQE), superficial conductivity, detectivity (D^*^), employed for hybrid materials Ti_3_C_2_T_ZnP_ and Ti_3_CNT_ZnP_ are shown below, as **Equation S6**$\boldsymbol{-}$**S**$\boldsymbol{10}$ respectively.

*Photo-response* $(R(\%))$

((Equation)) (S6)

$$R(\%)=\frac{{I_{Light}-I}_{dark}}{I_{dark}}x100$$

Where $I_{Light}$ and $I_{dark}$ are the current between the drain and source under light and dark illumination, respectively.

*Responsivity (*$\boldsymbol{R}_{\boldsymbol{\lambda}}\boldsymbol{)}$

((Equation)) (S7)

$$R_{\lambda}=\frac{{I_{Light}-I}_{dark}}{AP_{i}}$$

$\mathrm{Where}$ $P_{i}$ is the incident power density, and “*A”* is the active area of the photodetector. “*A”* is also the channel can be calculated from dimensions of electrodes (50 μm channel width and 100 μm length)

*External Quantum Efficiency* $(EQE (\%))$

((Equation)) (S8)

$$EQE (\%)\approx100x\frac{1240\times R_{\lambda}}{\lambda}$$

Where $\lambda$ is the wavelength of the light source excitation.

*Specific Detectivity (*$D^{*})$

((Equation)) (S9)

$$D^{*}\approx\frac{R_{\lambda}\sqrt{A}}{\sqrt{2\times q\times I_{dark}\times\Delta f}}$$

Where q is the elemental charge of electron.

Where q is the elemental charge of electron and $\Delta f$ is the bandwidth of the instrument. $\Delta f=\frac{1}{2\times t_{m}}$ where $t_{m}$ is the interval between measurements, based on a 0.5 second integration time set.

*Superficial Conductivity (*$\sigma_{sq})$

((Equation)) (S10)

$$\sigma_{sq}=\frac{LxI_{dark}}{WxV_{DS}}$$

Where L and W are the length and the width of the device, and $V_{DS}$is the drain-to-source voltage. Note that the material is extremely thin, and volumetric conductivity should not be applied. In any case, based on the height, the volumetric conductivity would yield values 2×10^8^ fold larger than the superficial conductivity.


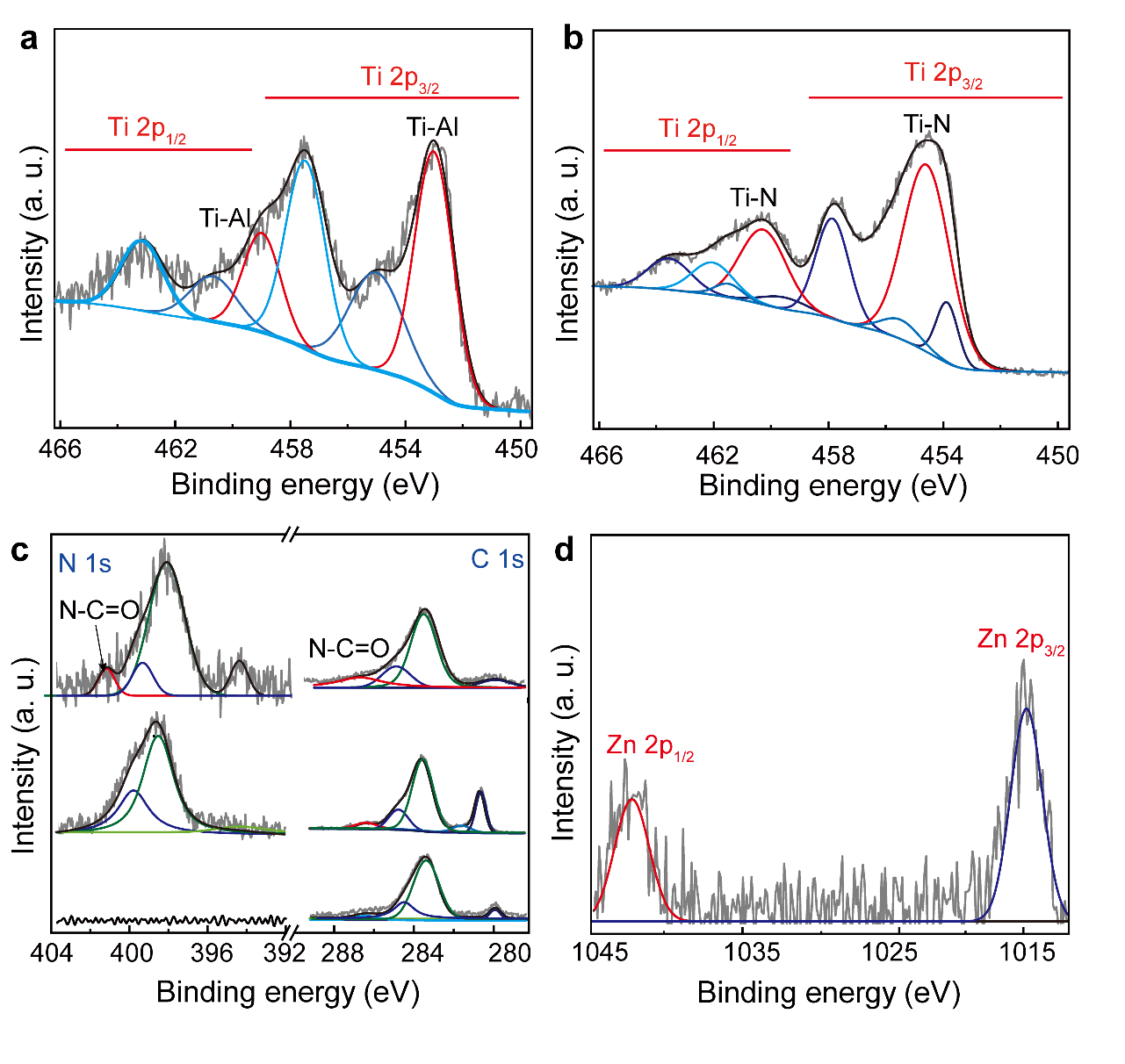


**Figure S1.** XPS spectra (a) Ti_2p_ of pristine Ti_3_AlC_2_ and (b) **a2**. (c) N_1s_ (left) and C_1s_ (right) of pristine Ti_3_AlC_2_, **a2**, **a3**. (d) Ti 2p Zn_2p_ of **a3**.


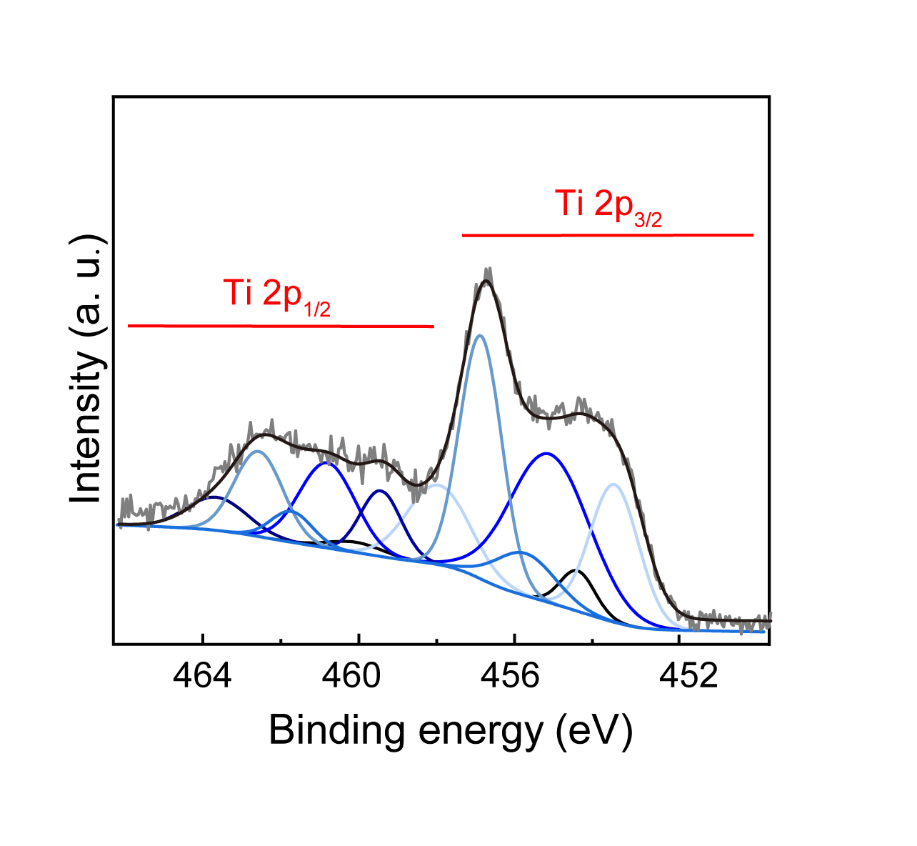


**Figure S2.** XPS Ti_2p_ of **b3**.


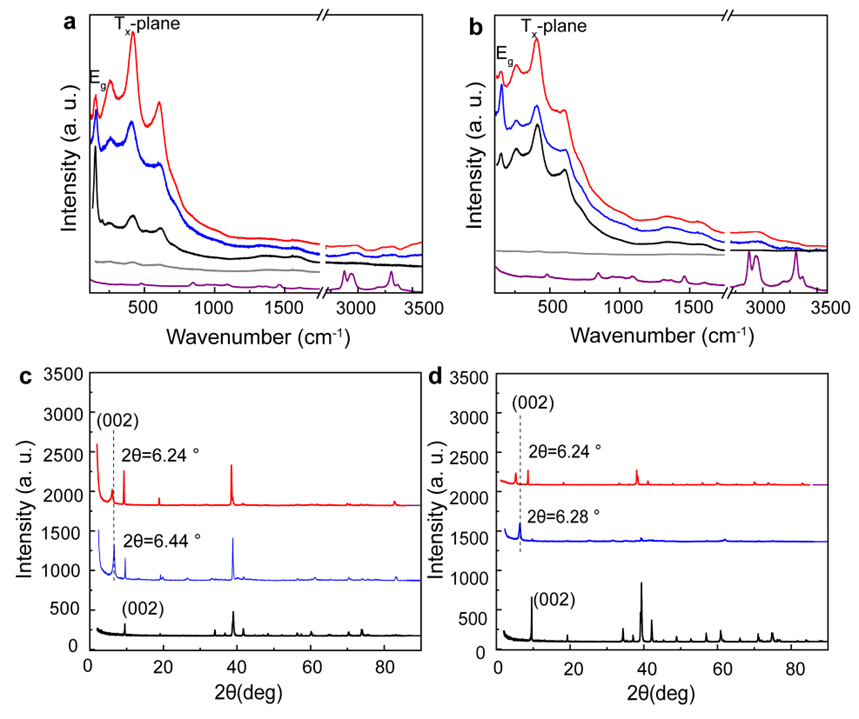


**Figure S3**. Raman spectra for (a) Ti_3_AlC_2_ (gray), **a1** (black), **a2** (blue), **a3** (red) and EDA (purple) and (b) Ti_3_AlCN (gray), **b1** (black), **b2** (blue) and **b3** (red), at 532 nm. XRD pattern (c) pristine Ti_3_AlC_2_ (black line), **a2** (blue line), **a3** (red line); (d) pristine Ti_3_AlCN (black line), **b2** (blue line), **b3** (red line).


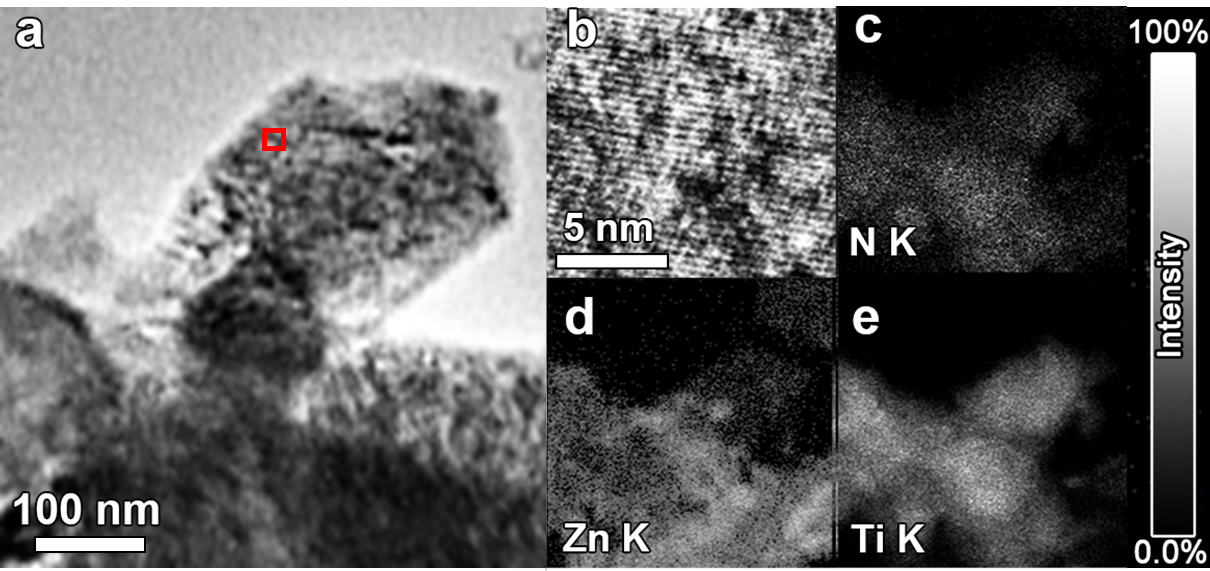


**Figure S4**. TEM (a) at low magnifications and (b) high magnifications for material **b3**. EDS of (c) N, (d) Zn and (e) Ti elements for **b3**, respectively.


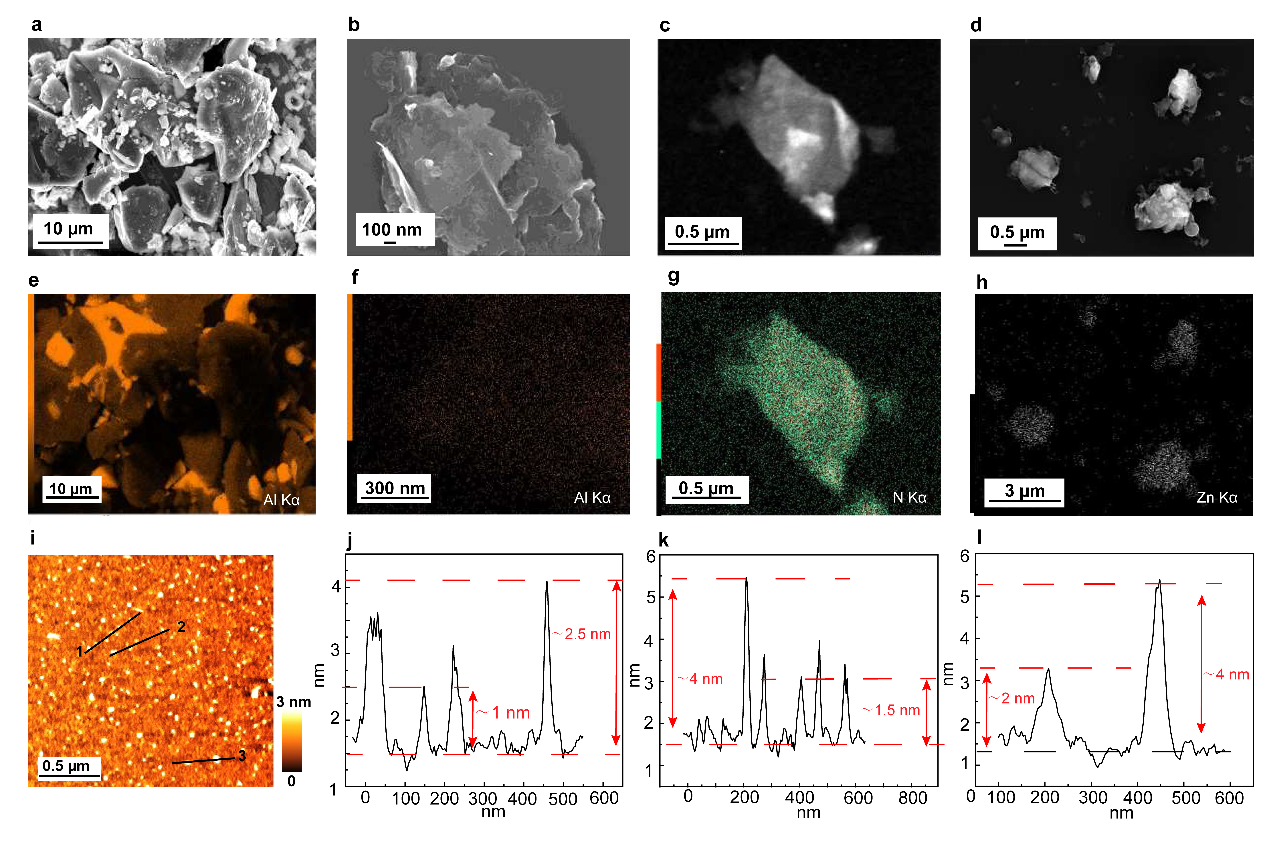


**Figure S5**. SEM images of (a) pristine Ti_3_AlC_2_, (b) **a1**, (c) **a2**, and (d) **a3**. EDS-SEM spectral mappings of (e) Al Kα for pristine Ti_3_AlC_2_, (f) Al Kα for **a1**, (g) N Kα for **a2**, and (h) Zn Kα for **a3**. (i) AFM image of a representative nanosheet **a3** and its line profile thickness, labeled as (j) *1*, (k) *2*, and (l) *3*.


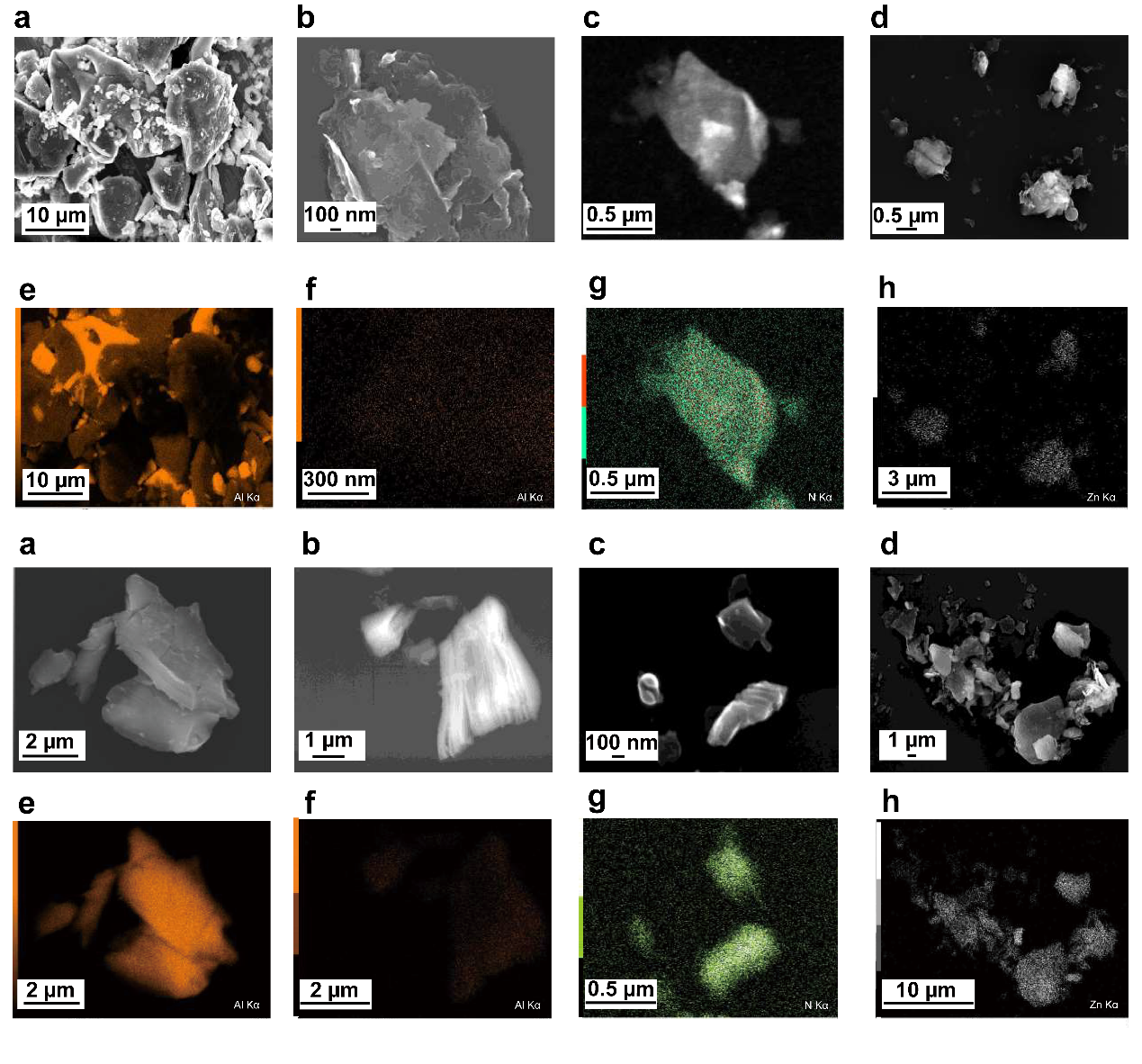


**Figure S6**. SEM images of (a) pristine Ti_3_AlCN, (b) **b1**, (c) **b2**, and (d) **b3**. EDS-SEM spectral mappings of (e) Al Kα for pristine Ti_3_AlCN, (f) Al Kα for **b1**, (g) N Kα for **b2**, and (h) Zn Kα for **b3**.

**Table S1.** List of CV reductions and oxidation values, in volts. CV was performed in acetonitrile using tetrabutylammonium hexafluorophosphate (TBAPF_6_) 0.1 M as the electrolyte. The working, reference, and counter electrodes were glassy carbon, Pt-mesh, and Pt-wire, respectively, with a scan rate of 25 mV·s^‒1^.

|  | Reduction^2^ | Reduction^1^ | Oxidation^1^ | Oxidation^2^ | Oxidation^3^ | Oxidation^4^ |
| --- | --- | --- | --- | --- | --- | --- |
| ZnP | –2.13 | –1.55 | × | 0.58 | 0.82 | 1.00 |
| **a2** | –1.39 | –1.02 | 0.053 | × | × | × |
| **a3** | –1.80 | –1.27 | 0.16 | 0.48 | 1.03 | × |
| **b2** | –1.71 | –1.15 | 0.32 | × | × | × |
| **b3** | –1.35 | –0.99 | 1.15 | 1.63 | × | × |


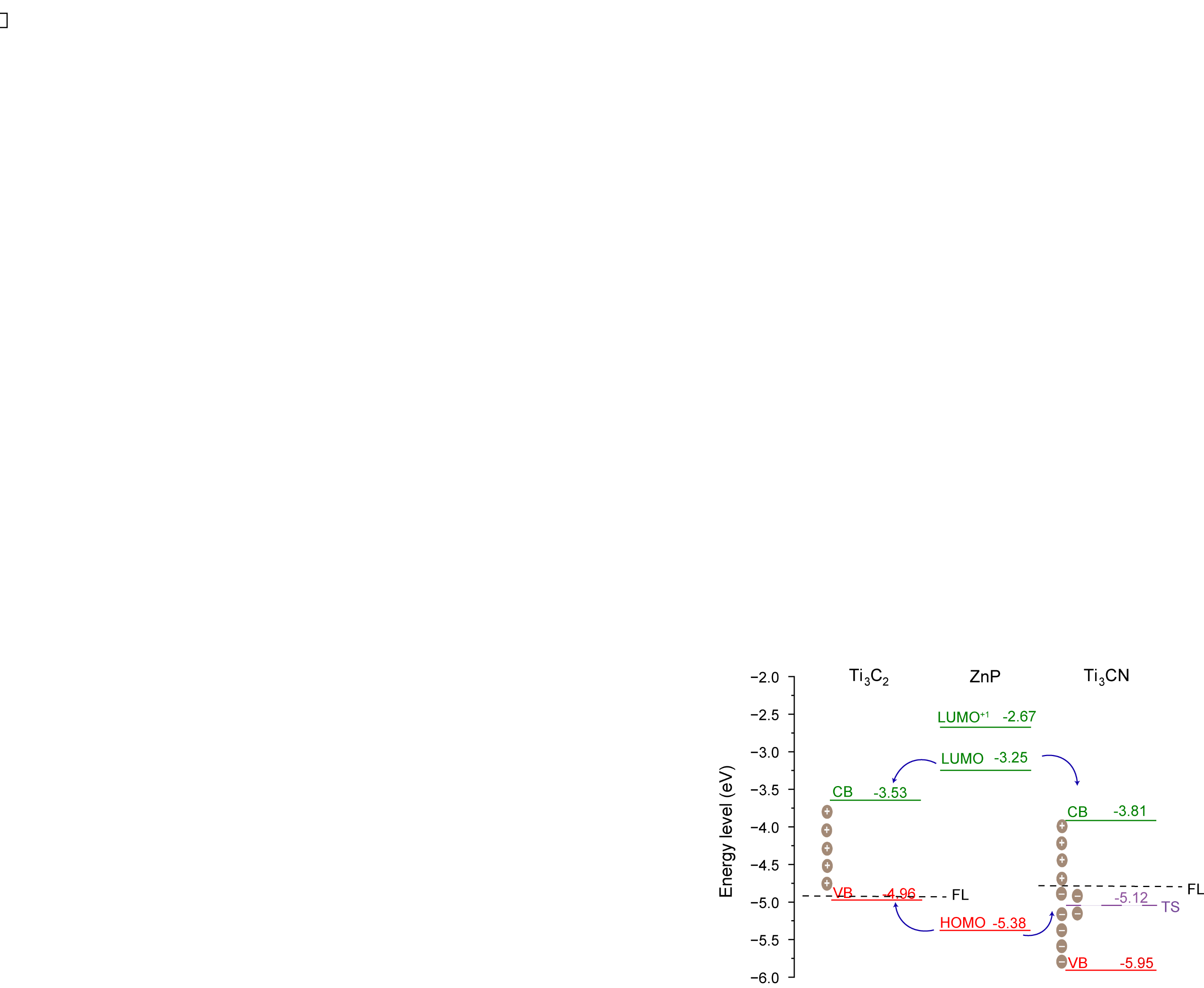


**Figure S7**. Energy diagram for ZnP, Ti_3_C_2_ and Ti_3_CN within **a3** and **b3** obtained by cyclic voltammetry. Note CB, VB, FL and TS corresponds to the conduction band minimum, valence band maximum, fermi level of the Fc/Fc^+^ pair (–4.8 eV), and trap states.


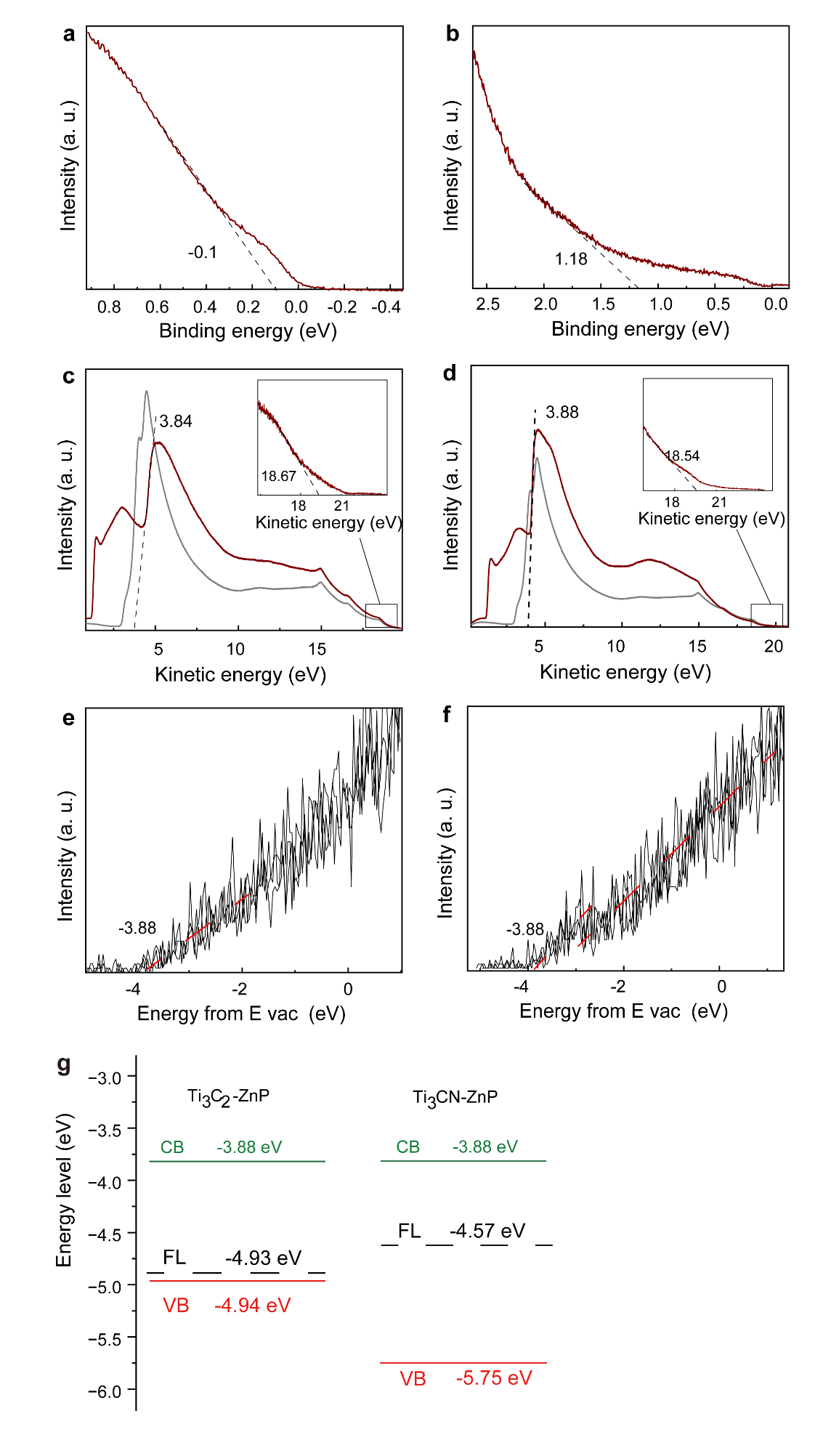


**Figure S8.** Binding energy for (a) **a3** and (b) **b3** and kinetic energy for (c) **a3** and (d) **b3** obtained towards UPS. LEIPS of (e) **a3** and (f) **b3**. (g) Energy diagram for ZnP, Ti_3_C_2_ and Ti_3_CN within **a3** and **b3** obtained by UPS/LEIPS.


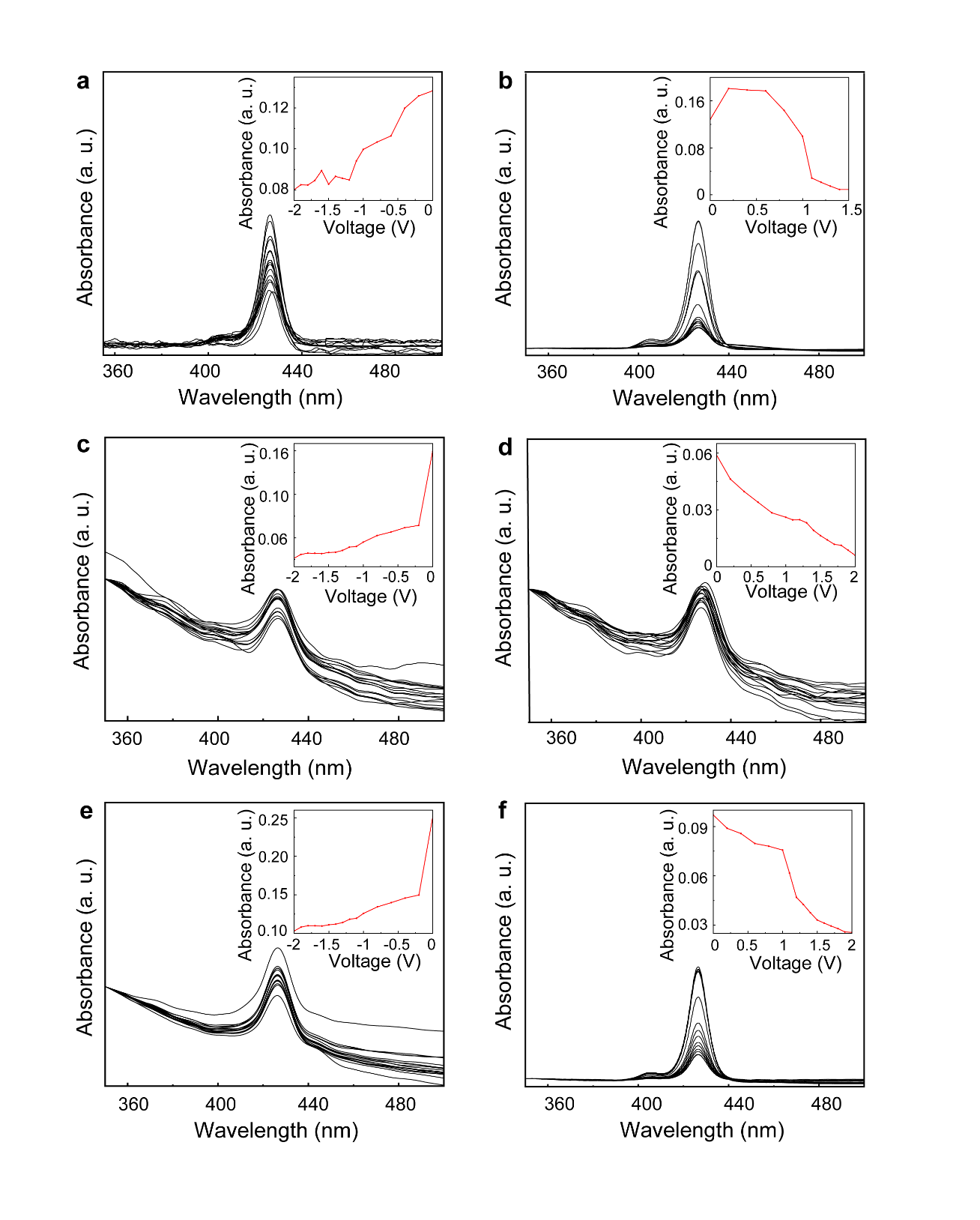


**Figure S9.** UV-vis absorbance under crescent voltages from 0 V to 2 V for (a) free ZnP, (c) **a3** and (e) **b3** and decrescent from 0 V to –2 V, for (b) free ZnP, (d) **a3** and (f) **b3** and increscent from 0 V to 2 V, in DMAc by employing 0.1 M of TBAPF_6_.


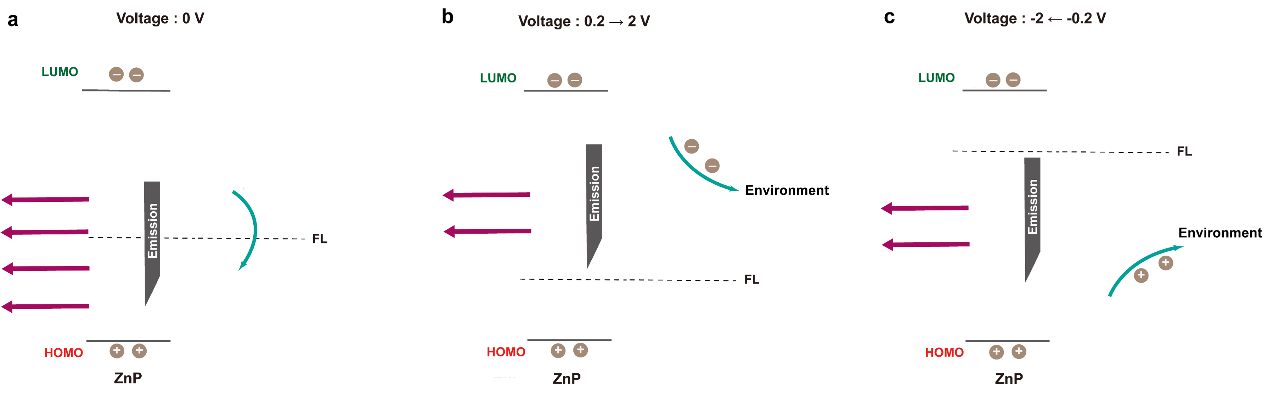


**Figure S10**. Carrier transfer process from free ZnP to the environment under (a) 0V, (b) positive and (c) negative voltage.


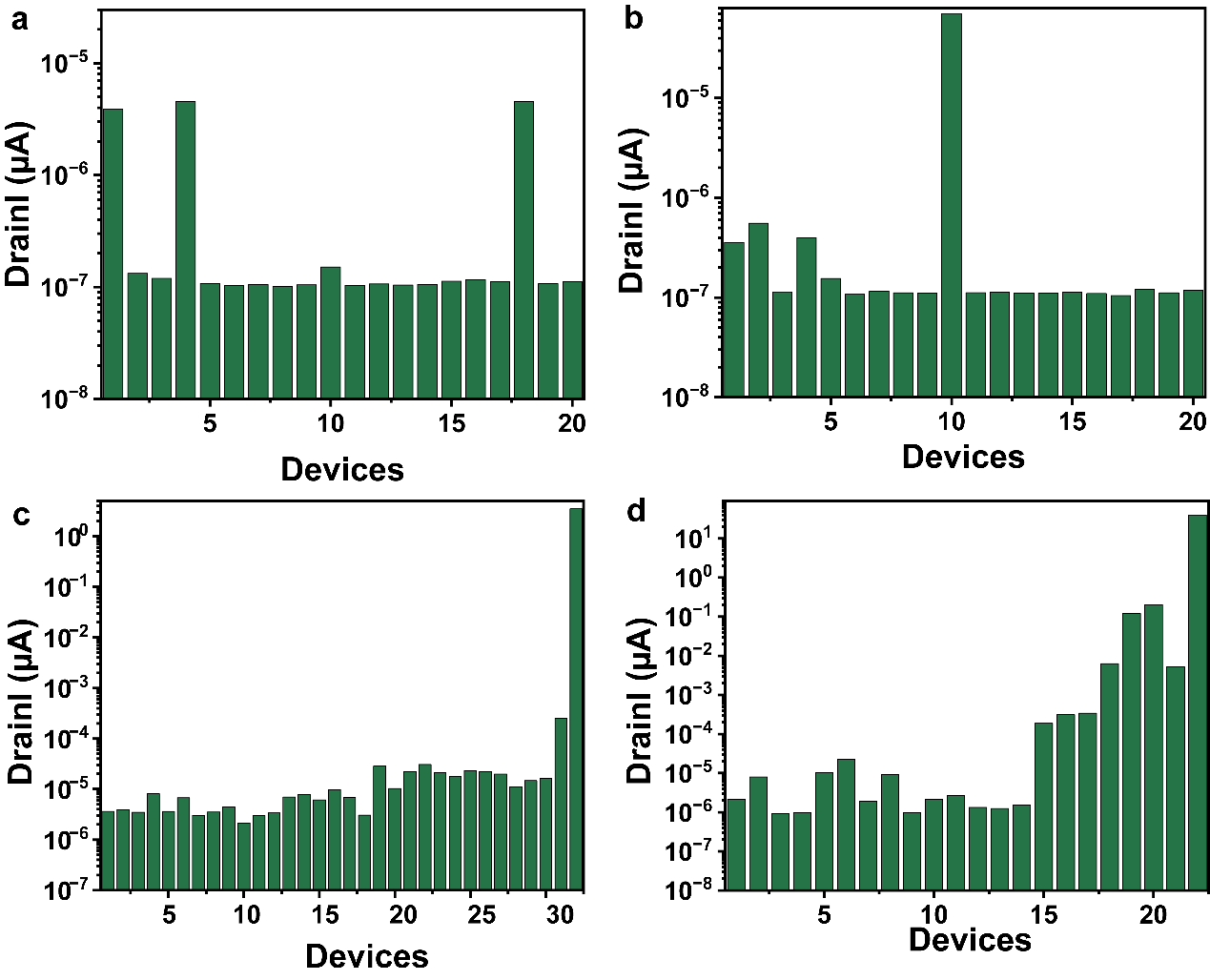


**Figure S11.** Performance comparison between different devices for (a) **a2**, (b) **b2**, (c) **a3** and (d) **b3**. All the experiments were carried out at room temperature, in vacuo, with a drain-to-source of 5 V, and in dark conditions. The value of the current in μA was 200 times larger than the than the superficial conductivity in μS·sq^‒1^.


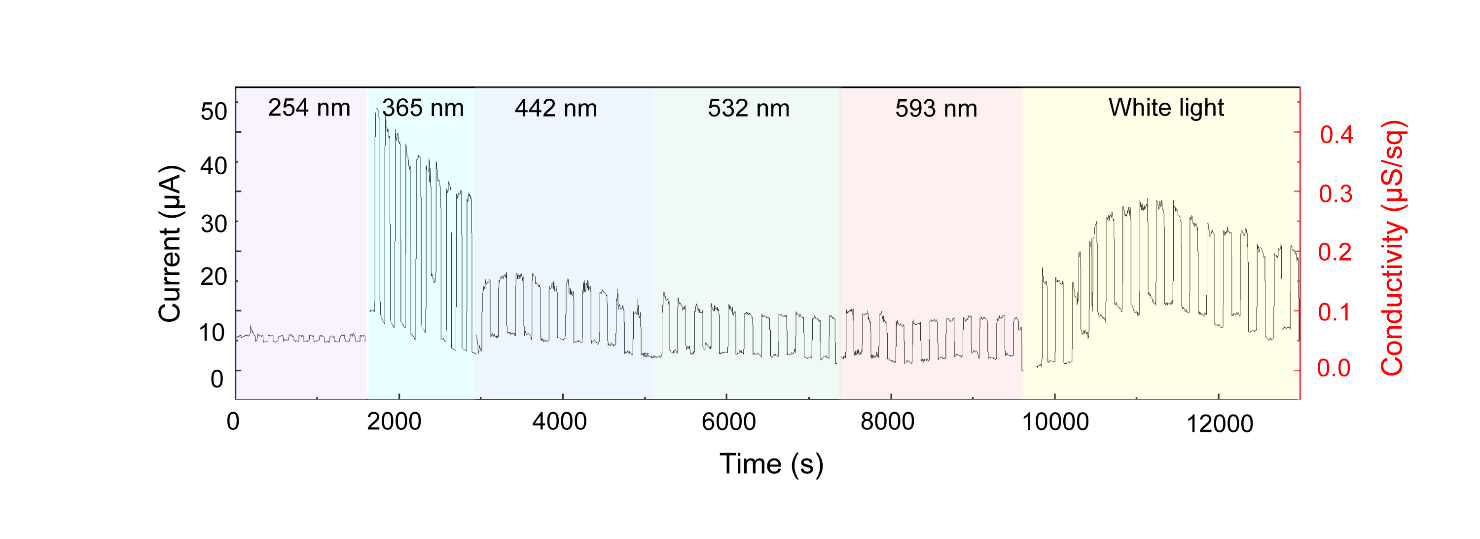


**Figure S12**. Performance of **b3** devices under different light-wavelength irradiation.


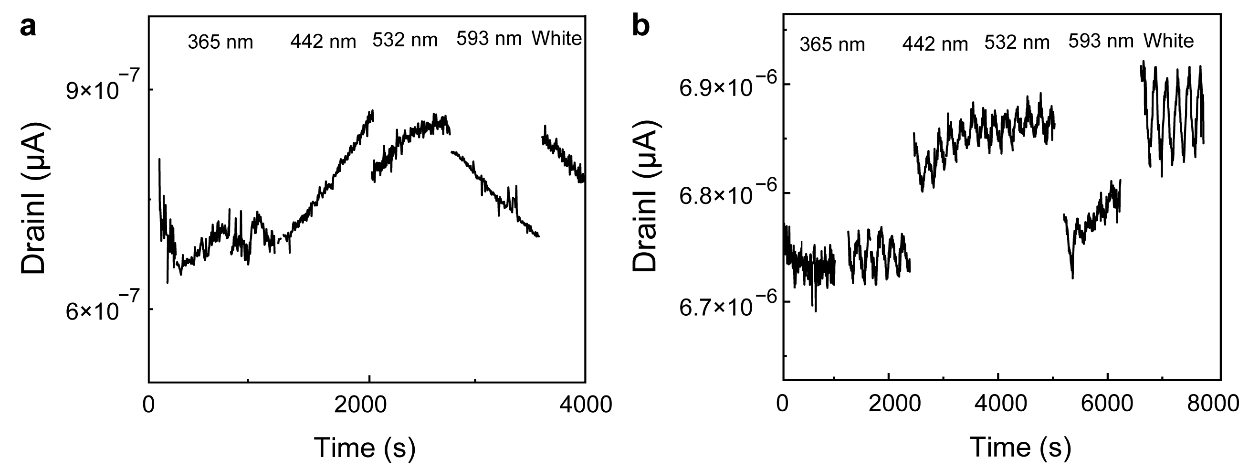


**Figure S13**. Performance of (a) **a2** and (b) **b2** devices under different light-wavelength irradiation.


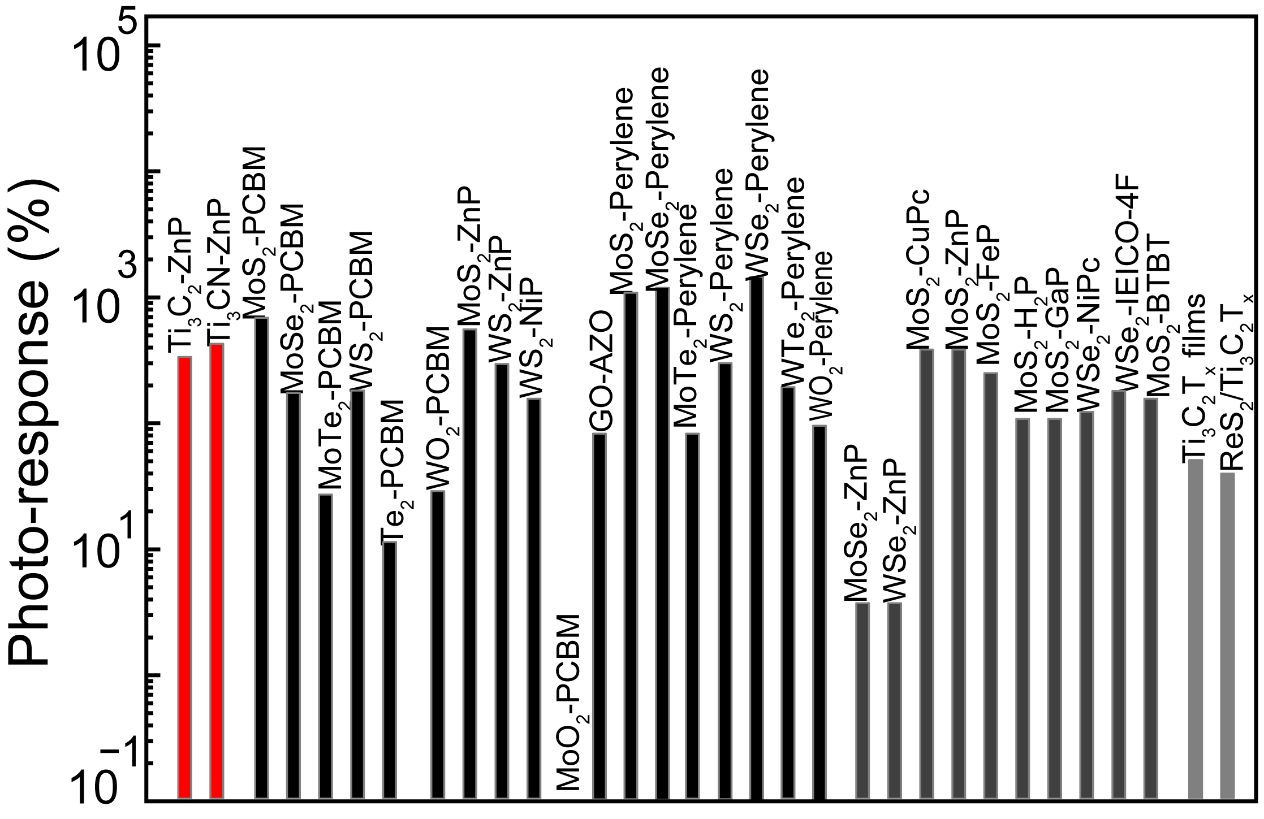


**Figure S14.** Comparison of the photo-response of **a3** and **b3** with other research results. Bibliography^[35,44,66,78-81,102]^ in the manuscript.
